# Precursor-Mediated Colloidal Synthesis of Compositionally Tunable Cu–Sb–M–S (M = Zn, Co, and Ni) Nanocrystals and Their Transport Properties

**DOI:** 10.1021/acs.chemmater.2c02605

**Published:** 2022-11-21

**Authors:** Maria Zubair, Vasily A. Lebedev, Mohini Mishra, Temilade Esther Adegoke, Ibrahim Saana Amiinu, Yu Zhang, Andreu Cabot, Shalini Singh, Kevin M. Ryan

**Affiliations:** † Department of Chemical Sciences and Bernal Institute, University of Limerick, V94 T9PX Limerick, Ireland; ‡ Catalonia Institute for Energy Research (IREC), 08930 Barcelona, Spain; § Catalan Institution for Research and Advanced Studies (ICREA), Passeig de Lluís Companys 23, 08010 Barcelona, Spain

## Abstract

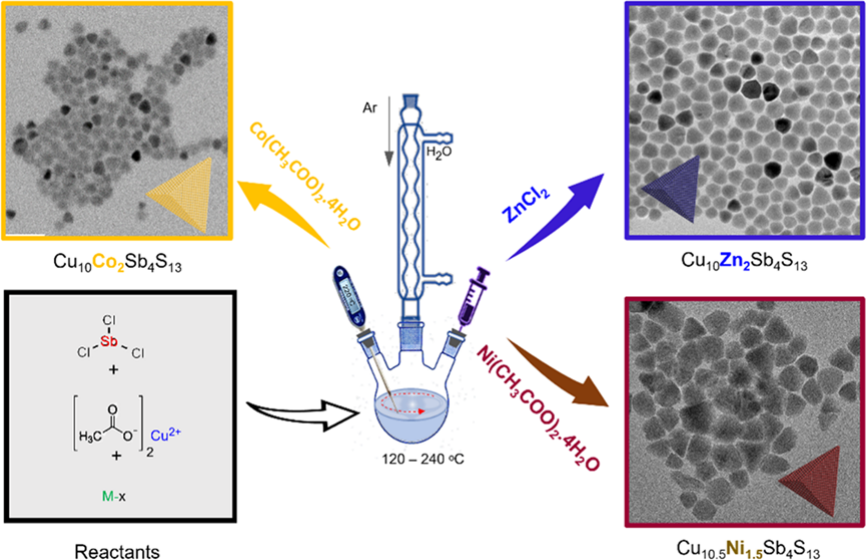

The solution-based colloidal synthesis of multinary semiconductor compositions has allowed the design of new inorganic materials impacting a large variety of applications. Yet there are certain compositions that have remained elusive—particularly quaternary structures of transition metal-based (e.g., Co, Zn, Ni, Fe, Mn, and Cr) copper antimony chalcogenides. These are widely sought for tuning the electrical and thermal conductivity as a function of the size, composition, and crystal phase. In this work, a facile hot injection approach for the synthesis of three different tetrahedrite-substituted nanocrystals (NCs) (Cu_10_Zn_2_Sb_4_S_13_, Cu_10_Co_2_Sb_4_S_13_, and Cu_10_Ni_1.5_Sb_4_S_13_) and their growth mechanisms are investigated. We reveal that the interplay between the Zn, Ni, and Co precursors on the basis of thiophilicity is key to obtaining pure phase NCs with controlled size and shape. While all of the synthesized crystal phases display outstanding low thermal conductivity, the Cu_10.5_Sb_4_Ni_1.5_S_13_ system shows the most enhanced electrical conductivity compared to Cu_10_Zn_2_Sb_4_S_13_ and Cu_10_Co_2_Sb_4_S_13_. This study highlights an effective synthesis strategy for the growth of complex quaternary nanocrystals and their high potential for application in thermoelectrics.

## 1 Introduction

Copper-based multinary colloidal nanocrystals (NCs) comprising low hazardous and earth-abundant elements are important due to their composition and size-tunable bandgap combined with their high light absorption coefficient, which make them relevant for electrocatalysis, photovoltaics, and thermoelectrics.^1−7^ The colloidal hot injection method has emerged as one of the most adaptable bottom-up synthesis techniques for tailoring the structure–property relationship of colloidal NCs for various applications with respect to the composition, size, shape, crystal phase, and cation or anion ratios.^3,7−17^ While extending this hot injection strategy to multinary copper-based chalcogenides is challenging due to the additional metal cations, many successful protocols have been reported for tuning composition in a wide variety of I–III–VI (CIS, CISe, and CIGSe) and I–IV–VI (CZTS and CZTSe) systems.^18−21^ Recently, Cu–Sb–S systems have gained interest, particularly due to the complexity of the crystal phases and potential opportunities to tune their thermal and electrical conductivity based on the crystal structure, shape, and size of nanocrystals.^22−26^ Four crystallographic phases of the Cu–Sb–S system exist, including tetrahedrite (Cu_12_Sb_4_S_13_), chalcostibite (CuSbS_2_), skinnerite (Cu_3_SbS_3_), and fematinite (Cu_3_SbS_4_). Among these phases, tetrahedrite, due to its naturally low-lattice thermal conductivity, is a promising alternative material for thermoelectric applications. Tetrahedrite with the chemical formula (Cu^+^)_10_(Cu^2+^)_2_Sb_4_S_13_ has both Cu(I) and Cu(II) ions in the crystal structure. Metal cations in the crystal structure of tetrahedrite with oxidation states +1, +2, and +3 allow versatility in the inclusion of various isovalent dopants. Optimum thermoelectric, optical, and magnetic properties require the partial substitution of other elements by the replacement of Cu, Sb, and chalcogen sites in Cu_12–*x*_A_*x*_Sb_4–*y*_B_*y*_S_13–*z*_Se_*z*_ (A = M^2+^; B = M^3+^).^27,28^ Substituted tetrahedrites are usually synthesized by solid-state reactions, which typically need longer reaction times (>30 h) and high reaction temperatures (>850 °C) to obtain high-quality bulk materials.^29,30^ In contrast, a limited number of approaches are reported with a solution-based synthetic approach. This includes solution-based polyol processes for the synthesis of pure and substituted tetrahedrite (50–200 nm) using reducing agents such as NaBH_4_.^31−34^ The fast injection of single source precursors followed by rapid cooling was also observed to be a generic approach for pure and substituted tetrahedrite synthesis, where the reaction was governed predominantly by thermodynamic control.^26^ The solution state synthesis of tetrahedrite substituted with an additional transition metal (Zn, Ni, Co, Mn, and Fe) is further complicated by the cross-nucleation and/or formation of unwanted side products because of a narrow thermodynamic window, making it difficult to synthesize stoichiometric compounds with desired phases using readily available precursors.

Herein, we report a facile approach for the synthesis of compositionally tunable Cu_10_Zn_2_Sb_4_S_13_, Cu_10_Co_2_Sb_4_S_13_, and Cu_10_Ni_1.5_Sb_4_S_13_ colloidal NCs. The NCs are produced by reacting metal salt precursors and tertiary dodecyl mercaptan (t-DDT) in the presence of 1-octadecene (ODE) and oleylamine (OLA). The coordinating solvent (OLA) facilitates the formation of an intermediate with the metal species and allows control of the NC size by selectively passivating the surface of the nucleated product. This circumvents the need for a separate reducing agent and ligands for the reaction. We observed that balancing the reactivity of Cu precursors with transition metals (Ni, Co, and Zn) is crucial to control the path of the reaction and to produce pure phase NCs with low polydispersity. Furthermore, the transport properties of these substituted nanostructures were measured in a temperature range from ambient to 490 °C. While all of the produced complex materials exhibit very low thermal conductivities, Cu_10.5_Ni_1.5_Sb_4_S_13_ further displays promising electrical conductivity, which makes it suitable for thermoelectric applications. Our results show that the transport properties of these NCs can be further enhanced by optimizing the level of substitution of the transition metal in the crystal structure of tetrahedrite (Cu_12–*x*_Sb_4_S_13_).

## 2 Experimental Section

### 2.1 Chemicals

Copper(II) acetate (Cu(CH_3_COO)_2_, 97%), antimony chloride (Sb(III)Cl, 99%), zinc(II) chloride, nickel(II) acetate tetrahydrate (Ni(CH_3_COO)_2_·4H_2_O), cobalt (II) acetate tetrahydrate (Co(CH_3_COO)_2_·4H_2_O), oleylamine(OLA), 1-octadecene (ODE), *t*-dodecyl mercaptan (*t*-DDT) anhydrous hexane, and acetone were all purchased from Sigma-Aldrich and used as received without further purification.

### 2.2 Synthesis of Cu_12–*x*_Sb_4_M*_x_*S_13_ (M = Zn, Ni, and Co) Nanocrystals

In a typical synthesis, 0.3 mmol Cu(CH_3_COO)_2_, 0.8 mmol Sb(Cl)_3_, and 0.1 mmol metal salt (ZnCl_2_, Ni (CH_3_COO)_2_·4H_2_O, or Co (CH_3_COO)_2_·4H_2_O) with a 4:1 ODE/OLA ratio by volume were added in a three-neck flask (25 mL) under an Ar atmosphere and connected to a Schlenk line via a condenser. The reaction mixture was evacuated for 40 min at 120 °C to form a clear metal–ligand complex and to remove any moisture content. During heating, the color of the reaction mixture changed from dark blue to green at ≈80 °C and finally to brownish orange at 120 °C. Based on this color change, the dissolution of precursors in the solvent can be described as a reduction of Cu^2+^ to Cu^1+^ followed by the dissolution of Sb and Zn/Ni or Co. The reducing solvent oleylamine was used for the reduction of Cu^2+^ to Cu^1+^ in the reaction system. To produce a reducing environment, oleylamine forms a complex [Cu(OLA)_2_] with the cation (Cu^2+^), as was previously observed in the literature.^35,36^ Although the copper cation in this complex is chelated, a dissociative-interchange mechanism constantly exchanges the ligands with oleylamine from the solvent, leaving Cu^2+^ available for the nucleophilic attack of the active sulfur source in the form of hydrogen sulfide.^36^ After this, Ar was purged in the solution, the temperature was increased to 240 °C, and 5 mmol *t*-DDT was swiftly injected, which turned the solution color into reddish brown in the case of Zn substitution and black for Ni and Co. The reaction was then allowed to proceed with continuous stirring for 15 min at 240 °C for the growth of the nanocrystals. By removing the heating mantle, the reaction was stopped and the dark solution was quenched with 5 mL of anhydrous toluene after being allowed to cool down to 100 °C. The obtained nanocrystals were isolated from the solution by centrifugation, and to effectively purify the nanocrystals, the mother liquor was divided into two 50 mL centrifuge tubes dispersed in 10 mL of hexane and centrifuged at 5000 rpm for 3 min. The remaining material was redispersed in hexane and acetone (3:1 v/v) and centrifuged for 5 min at 3000 rpm, following which the supernatant was discarded. After that, numerous precipitation and dispersion cycles employing chloroform/acetone/hexane (1:1:2 v/v) were used to further purify the semipure nanocrystals. The synthetic protocol for pure tetrahedrite (Cu_10_Sb_4_S_13_) is provided in the Supporting Information.

### 2.3 Materials Characterization

#### 2.3.1 Electron Microscopy

Transmission electron microscopy (TEM) and angular dark-field scanning transmission electron microscopy (STEM) were used to analyze the structure of the NCs on a JEOL JEM-2011F that was run at an accelerating voltage of 200 kV and mounted with a Gatan camera. Prior to imaging, samples were drop-casted on a nickel TEM grid. Using ImageJ software and counting >100 particles per sample, size statistics were calculated. On an FEI Titan Cubed Themis G2 300, aberration-corrected microscopy, high-resolution TEM (HRTEM) imaging, high-angle annular dark-field STEM (HAADF-STEM) imaging, and energy-dispersive X-ray spectroscopy (EDX) with elemental mapping were carried out.

#### 2.3.2 X-Ray Diffraction (XRD) Analysis

The XRD patterns of samples were acquired on an EMPYREAN with Cu Kα radiation and a one-dimensional Lynxeye detector. The sample was washed with isopropanol and acetone and then drop-casted on a glass slide for XRD analysis.

#### 2.3.3 Raman Spectroscopy

A 532 and 632.8 nm laser in an NT-MDT instrument mounted with NTEGRA spectra was used to gather the Raman data. The laser power of 2.5 mW and 20 s of integration times with 10 accumulations were used to get all spectra.

#### 2.3.4 X-Ray Photoelectron Spectroscopy

With the help of a Kratos Axis Ultra spectrometer, the NCs XPS data were collected. However, utilizing monochromated Al K radiation with an energy of 1486.6 eV at 20 eV, high-resolution spectra were recorded. A mixed Gaussian–Lorentzian function with a Shirley-type background subtraction was utilized for peak synthesis. Low-energy electrons were showered onto the samples to effectively neutralize the charge. Using the charge reference value of C 1s at 284.8 eV, binding energies (BE) were calculated. The sample was dropped onto a glass slide for drying under argon before being analyzed.

#### 2.3.5 Inductively Coupled Plasma Mass Spectroscopy (ICP-MS)

For ICP-MS measurements, the samples were prepared by dissolving 1 mg in 2 mL of 1% HNO_3_ at 80 °C for 2 h. The solution was digested overnight at room temperature and diluted to 50, 40, and 10 ppm concentrations for the ICP-MS analysis.

#### 2.3.6 Transport Properties

For transport property measurements, roughly 0.5 g of each NC composition was prepared. To remove the surface ligands, the NCs were washed thoroughly by several precipitation and dispersion steps until they remained insoluble. The washed NCs were vacuum-dried overnight in an oven at 70 °C before the measurement of the thermoelectric properties. The cleaned and dried nanoparticles were annealed at 490 °C and then hot-pressed into pellets. All of the measurements were carried out at temperatures below 490 °C.

## 3 Results

**Figure 1:**
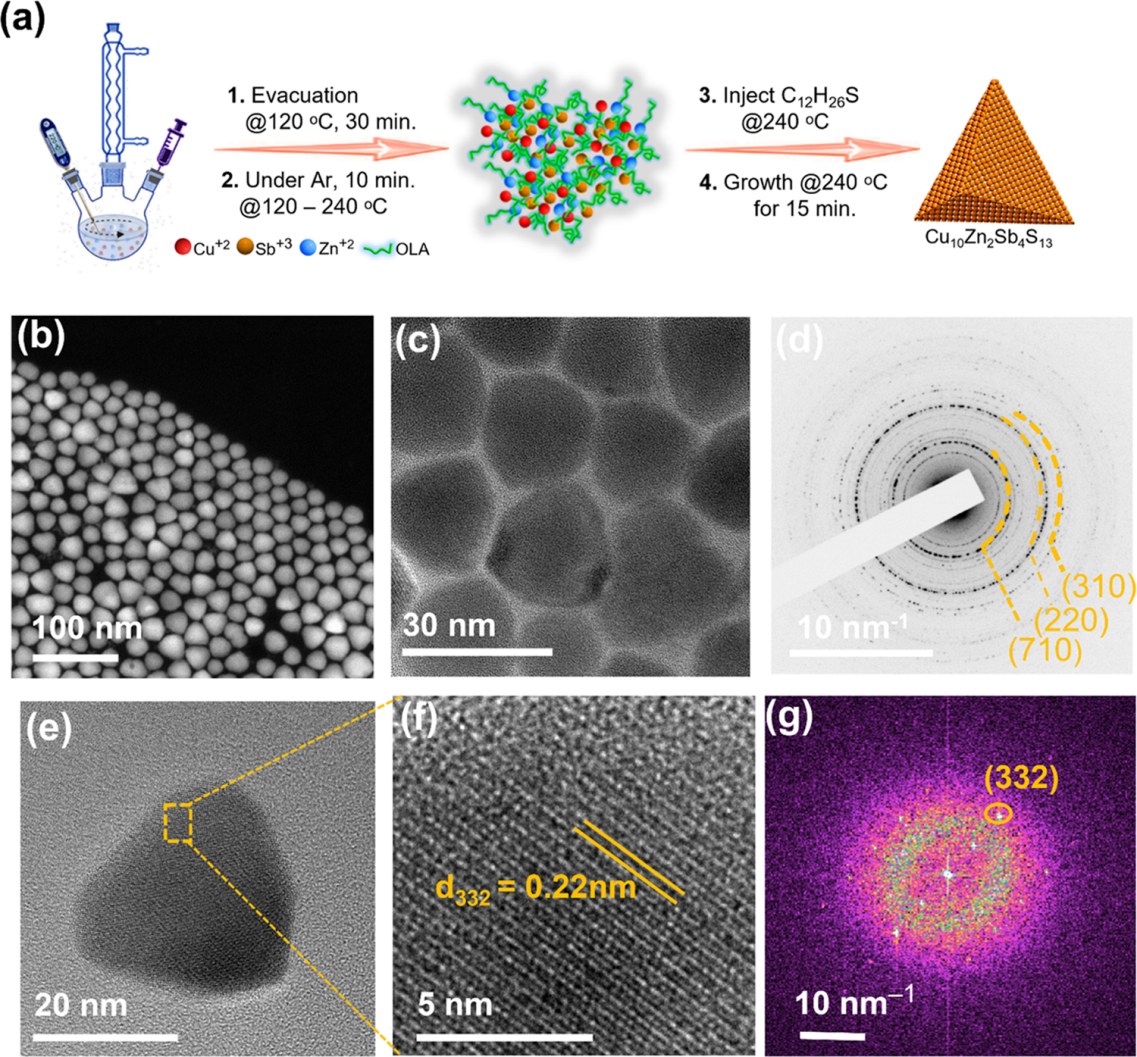
(a) Schematic for the synthesis of Cu_10_Zn_2_Sb_4_S_13_ NCs. (b) Dark-field and (c) bright-field TEM images of cubic phase Cu_10_Zn_2_Sb_4_S_13_ NCs. (d) Selected area electron diffraction (SAED) pattern; (e) HRTEM image of a single cubic Cu_10_Zn_2_Sb_4_S_13_ nanocrystal with (f) the magnified image of the selected area marked by the dashed line in panel (e) along with (g) fast Fourier transform (FFT).

The synthesis protocol for the formation of the tetrahedrite-substituted NCs (Cu_10_Zn_2_Sb_4_S_13_, Cu_10_Co_2_Sb_4_S_13_, Cu_10_Ni_1.5_Sb_4_S_13_), as shown in Figure 1a, involves the use of Cu(II) acetate, SbCl_3_, and metal salts (ZnCl_2_, Co(CH_3_COO)_2_·4H_2_O and Ni(CH_3_COO)_2_·4H_2_O) in the initial stage. A sulfur precursor (*t*-DDT) is subsequently introduced via separate injection at 240 °C in the presence of 1-ODE as the noncoordinating solvent and oleylamine as the ligand. Figure 1b,c shows the dark- and bright-field TEM images of the Cu_10_Zn_2_Sb_4_S_13_ NCs. A histogram of Cu_10_Zn_2_Sb_4_S_13_ nanoparticles (Figure S1a) shows an average size of 30 nm. The selected area electron diffraction (SAED) pattern (Figure 1d) can be indexed to the cubic phase of Cu_10_Zn_2_Sb_4_S_13_. The high-resolution TEM (HRTEM) images, along with fast Fourier transform (FFT), also revealed the high crystallinity of the cubic phase of the particles. The HRTEM image of Cu_10_Zn_2_Sb_4_S_13_ NCs is shown in Figure 1e,f. The crystallinity of the NCs is confirmed by the uniformity of the lattice fringes, and FFT in Figure 1g was used to calculate the lattice spacing. The calculated *d*-spacing of 0.22 nm from Cu_10_Zn_2_Sb_4_S_13_ NCs corresponds to the (332) crystallographic plane.

**Figure 2:**
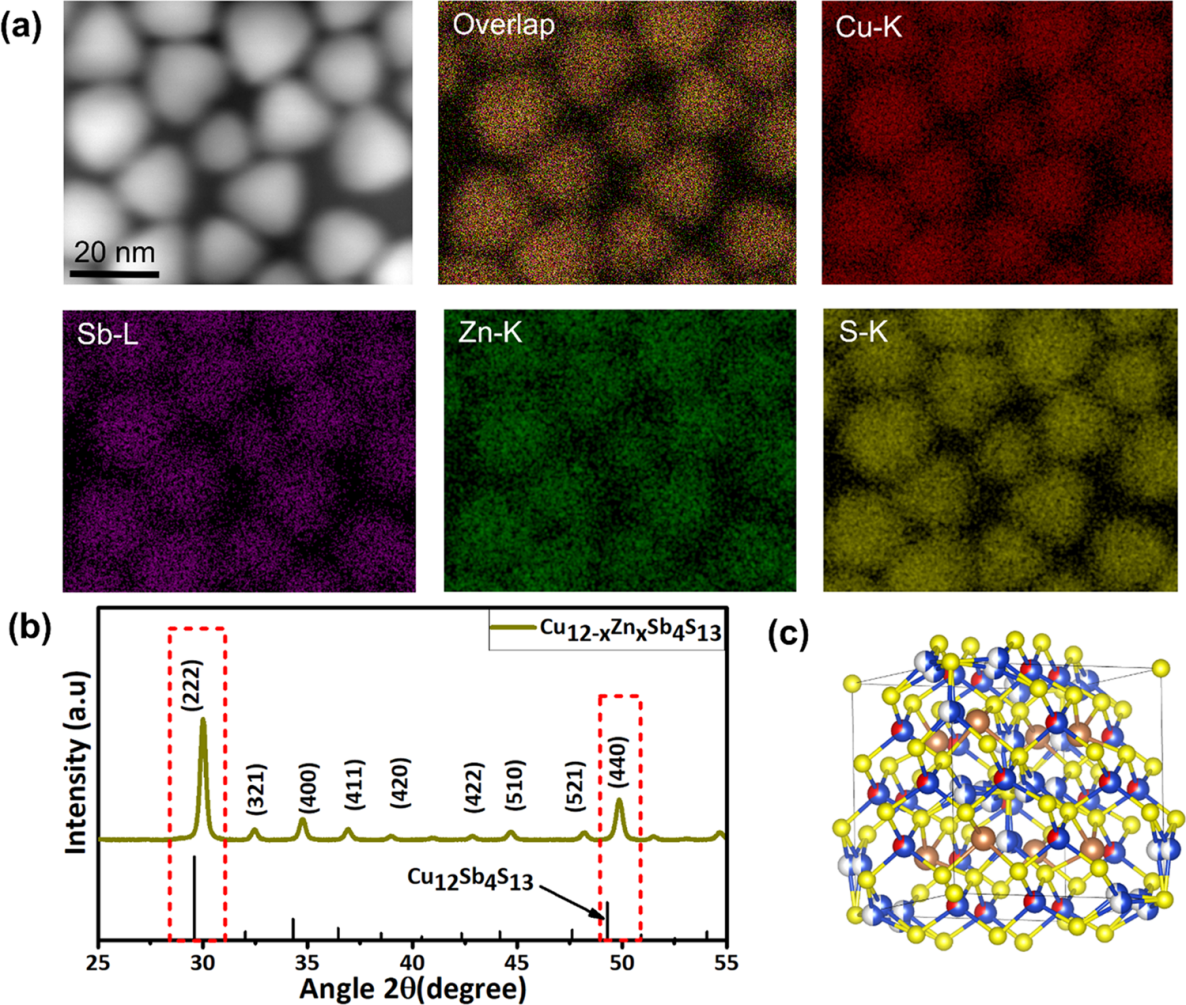
(a) HAADF-STEM image of Cu_10_Zn_2_Sb_4_S_13_ NCs with associated STEM-EDS maps of Cu (red), Zn (green), Sb (purple), and S (yellow). (b) XRD pattern of the Cu_10_Zn_2_Sb_4_S_13_ NCs. (c) Crystal structure model of tetrahedrite: blue (Cu^I^), yellow (S^II^), brown (Sb^III^), and red (M^II^).

The compositional and structural characterizations of Cu_10_Zn_2_Sb_4_S_13_ NCs were carried out with HAADF-STEM and XRD. Figure 2a shows the high-angle annular dark-field scanning transmission electron microscopy (HAADF-STEM) image with the corresponding EDX elemental mapping highlighting the uniform distribution of all of the elements in the Cu_10_Zn_2_Sb_4_S_13_ NCs. The crystal phase and composition were investigated using XRD (Figure 2b). The main diffraction peaks at 2θ = 29.59, 34.30, 49.29, 54.06, and 58.56° correspond to the planes (222), (400), (440), (611), and (622) of cubic tetrahedrite with the *I*-43*m* space group (JCPDS No. 01-086-4944). The displacement of Cu^2+^ (0.73 Å) with Zn^2+^ (0.60 Å) in the crystal lattice causes a slight shift of diffraction peaks toward higher 2θ values. Figure 2c shows the crystal structure model of Zn-substituted tetrahedrite. Rietveld refinement analysis of the XRD pattern of the NCs (Figure S2) confirms the cubic phase with the Cu_10_Zn_2_Sb_4_S_13_ crystal composition, further supporting the TEM observations (Supporting Information includes the crystal data and atomic coordinates).

**Figure 3:**
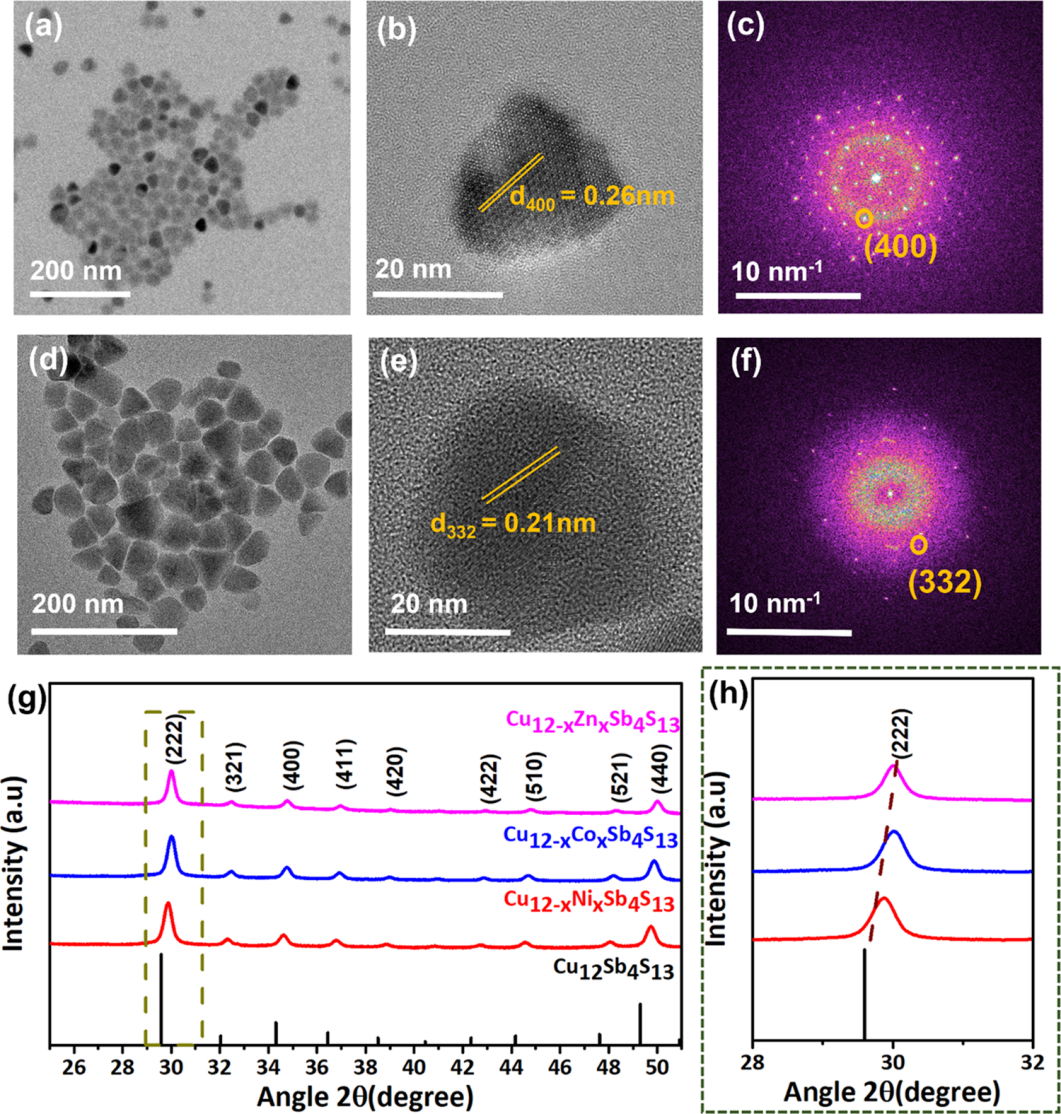
(a–c) Low-magnification TEM and HRTEM images of single Cu_10_Co_2_Sb_4_S_13_ NCs with fast Fourier transform (FFT). (d–f) Low-magnification TEM and HRTEM images of single cubic Cu_10.5_Ni_1.5_Sb_4_S_13_ NCs with fast Fourier transform (FFT) showing the presence of a cubic phase. (g) XRD patterns for NCs, along with simulated XRD patterns of tetrahedrite, where panel (h) shows the shift in (222) planes with the substitution of Ni (red), Co (blue), and Zn (pink).

For the synthesis of Cu_10_Co_2_Sb_4_S_13_ NCs, hydrated acetate of Co was combined with Cu (in acetate) and Sb (in halide form) with thiol as an S source. The synthesized Cu_10_Co_2_Sb_4_S_13_ NCs also exhibit a pyramidal shape with an average size of 30 nm (Figure 3a). The HRTEM image (Figure 3b) shows that Cu_10_Co_2_Sb_4_S_13_ NCs are crystalline with periodic lattice fringes spaced by 0.25 nm corresponding to the (400) planes (Figure 3c) of cubic Cu_10_Co_2_Sb_4_S_13_ NCs. As shown in Figure 3d, the Cu_10_Ni_1.5_Sb_4_S_13_ NCs with an average size of 50 nm were also synthesized with hydrated acetate of Ni. Figure 3e shows the HRTEM image of Cu_10_Ni_1.5_Sb_4_S_13_ NCs with a calculated *d*-spacing of 0.21 nm corresponding to the (332) plane (Figure 3f) of cubic phase Cu_10.5_Ni_1.5_Sb_4_S_13_ NCs. The size distribution histograms of these substituted NCs (Figure S1b,c) show that a good uniformity of NCs is achieved using this synthetic approach. The XRD patterns (Figure 3g) of all three nanostructures with Ni, Co, and Zn show patterns consistent with that of bulk tetrahedrite. However, the peak positions narrowly shifted to higher angles depending on the size of the substituted cation (Figure 3h).^37^ Raman spectra (Figure S3) of all of the synthesized NCs (Cu_10_Zn_2_Sb_4_S_13_, Cu_10_Co_2_Sb_4_S_13_, and Cu_10.5_Ni_1.5_Sb_4_S_13_) show a strong peak at 357 cm^–1^, which is a characteristic peak for tetrahedrite.^38^ The vibrations of M–S bonds result in a strong Raman peak, while the observed shift in the frequencies of these peaks is due to the difference in the force constant for different incoming cations. Since Cu^II^–S bonds are transformed to M^II^–S bonds (M = Zn, Co, and Ni) during the formation of substituted tetrahedrite, the bond strength change causes a red shift in the Raman position (357 cm^–1^). HAADF-STEM (Figure S4) with corresponding EDX elemental mapping shows a uniform distribution of all four elements in the Cu_10_Co_2_Sb_4_S_13_ NCs. X-ray photoelectron spectroscopy (XPS) analysis was performed, as shown in Figure S5, to confirm the oxidation states of the constituent elements in the NCs. In the Cu XPS spectrum, an intense set of doublet peaks at 951.52 eV for 2p_1/2_ and 931.75 eV for 2p_3/2_ were observed. The two peaks are separated by 19 eV, confirming the presence of a Cu^1+^ state. The absence of Cu^II^ peaks also confirms that M^II^ ions replaced the Cu^II^ ion in the tetrahedrite (Cu_12–*x*_^I^Cu^II^*_x_*Sb_4_S_13_) structure. Inductively coupled plasma mass spectroscopy (ICP-MS) results show (Table S1) that the *x* value for Co and Zn in Cu_12–*x*_M*_x_*Sb_4_S_13_ is ∼2 (i.e., Cu_10_Zn_2_Sb_4_S_13_, Cu_10_Sb_4_Co_2_S_13_), endorsing that the substituent bivalent ions replaced an equivalent amount of Cu^II^, while a value of 1.5 was observed for Ni (Cu_10_ Ni_1.5_ Sb_4_S_13_).

**Figure 4:**
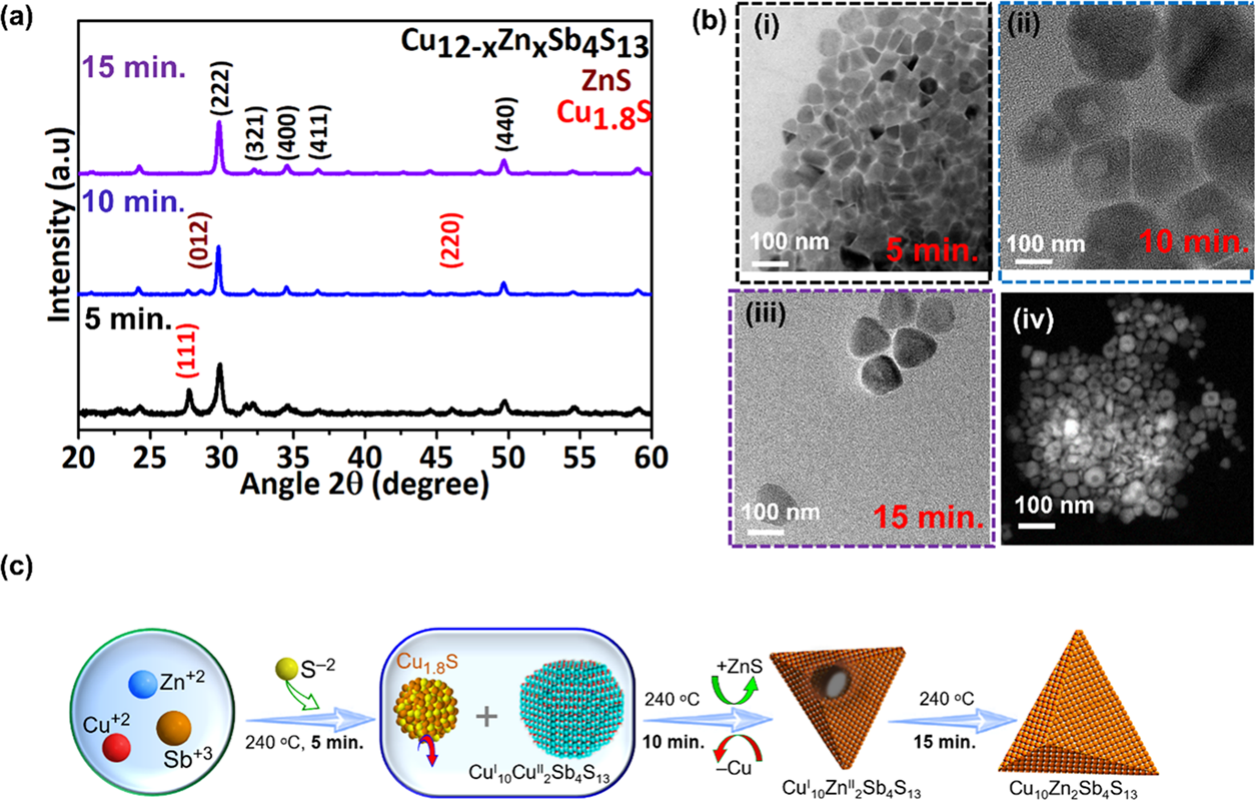
(a) XRD pattern of aliquots at different reaction times (5, 10, and 15 min) for cubic Cu_10_Zn_2_Sb_4_S_13_. (b) TEM of aliquots for the NC shape evolution at 5, 10, and 15 min (i–iii), and the STEM image (iv) of the aliquot at 10 min. (c) Schematic illustration of the evolution steps of Cu_10_Zn_2_Sb_4_S_13_ NCs at different reaction times.

We investigated the evolution of NCs during the formation of substituted tetrahedrite NCs (Cu_10_Zn_2_Sb_4_S_13_, Cu_10_Co_2_Sb_4_S_13_, and Cu_10.5_Ni_1.5_Sb_4_S_13_) to further evaluate the formation mechanism of these NCs with different metal precursors. Figure 4 collates the results for the NC evolution within the Cu_10_Zn_2_Sb_4_S_13_ crystal system. For the Cu_10_Zn_2_Sb_4_S_13_ system, the Cu_1.8_S phase coexists with the quaternary (Cu_10_Zn_2_Sb_4_S_13_) phase (Figure 4a) from 3 to 10 min upon the injection of the thiol source. After 5 min of injecting *t*-DDT, the peak intensity corresponding to Cu_1.8_S decreases and a small peak corresponding to ZnS emerges. The TEM analysis (Figure 4b) shows the presence of Cu_1.8_S hexagonal platelets at the initial stages after 5 min of reaction. Transformation of hollow particles to solid particles of average size 50 nm was observed for aliquots from 10 to 15 min. Two basic explanations have been put out to explain the mechanism of copper antimony sulfide-based nanocrystals: (i) inter-reaction theory and (ii) cation-exchange theory. According to the inter-reaction theory, the synthesis of copper-based ternary metal sulfides considers both the reaction between Cu_2_S and other binary metal sulfides in the reaction mixture and the reaction between chalcogen and the second metal. On the other hand, for the conventional cation-exchange theory, Cu*_x_*S preferentially forms in the early phases of the reaction based on the idea that copper ions are more reactive than those of other metals. In the cation-exchange reaction, the diffusion of foreign cations into the crystal structure is significantly influenced by the movement of the copper ion. Liang et al. reported Cu_12_Sb_4_S_13_ synthesis by the reaction of preformed Cu_1.8_S and Sb_2_S_3_.^39^ In the present report, we observe the presence of Cu_1.8_S at the initial stages with no evidence for the formation of Sb_2_S_3_, which suggested the sequential cation-exchange process with in situ formed Cu_1.8_S for the formation of Cu_10_Zn_2_Sb_4_S_13_ NCs instead of inter-reaction between binary sulfides. The morphology of the NCs changes from nanoplates to pyramid-shaped nanoparticles for Cu_10_Zn_2_Sb_4_S_13_ NCs with the incorporation of Zn. EDX analysis shows that nanocrystals formed after 5 min of reaction time are Zn deficient and have an elemental composition similar to (Cu^+^)_10_(Cu^2+^)_2_Sb_4_S_13_, while the sample collected after 10 min indicates incorporation of Zn via the partial cation-exchange mechanism with isovalent Cu^2+^ (Figure S7). The cation-exchange reactions that have been carried out above 230 °C for the synthesis of ternary metal chalcogenides with a reactive precursor of Zn would prefer heteroepitaxial formation of ZnS, according to earlier reports.^40^ However, a decrease in the reactivity of the precursor will permit cationic diffusion in addition to shell formation.^26,41^ In this study, we used ZnCl_2_ as a Zn source, and according to the HSAB theory, borderline (Zn^2+^ and Cl^–^) acid and base make ZnCl_2_ less reactive. This Zn precursor results in unequal diffusion between Cu^2+^ and Zn^2+^ on the similar S^2–^ anion frameworks resulting in the formation of hollow particles, as observed in the aliquot taken after 10 min, which further transformed to solid Cu_10_Zn_2_Sb_4_S_13_ NCs after 15 min of reaction time with complete cation exchange between Cu^2+^ and Zn^2+^. The evolution sequence of the Cu_10_Zn_2_Sb_4_S_13_ NCs at different reaction times is schematically depicted in Figure 4c. Based on the above observations, the most likely growth mechanism is cation exchange. The growth process starts with the thermal decomposition of the Cu precursor to form Cu*_x_*S, which is then transformed into (Cu^+^)_10_(Cu^2+^)_2_Sb_4_S_13_ with the incorporation of Sb^3+^. Gradually with time (5–15 min), diffusion of the transition metal (Zn^2+^) results in a quaternary composition. Interestingly, for Ni-substituted tetrahedrite, the formation of Cu_10.5_Ni_1.5_Sb_4_S_13_ NCs was detected just after 3 min of injecting *t*-DDT with the XRD pattern showing low-intensity peaks at 2θ = 27.6, 32.0, and 45.99° for the (111), (200), and (220) diffraction planes of Cu_1.8_S. The pure phase of Cu_10_._5_Ni_1.5_Sb_4_S_13_ NCs was ultimately obtained after only 7 min (Figure S8). The formation of quaternary Cu_10_Co_2_Sb_4_S_13_ NCs (Figure S9) was also observed within 5 min after the injection of the thiol source.

## 4 Discussion

**Figure 5:**
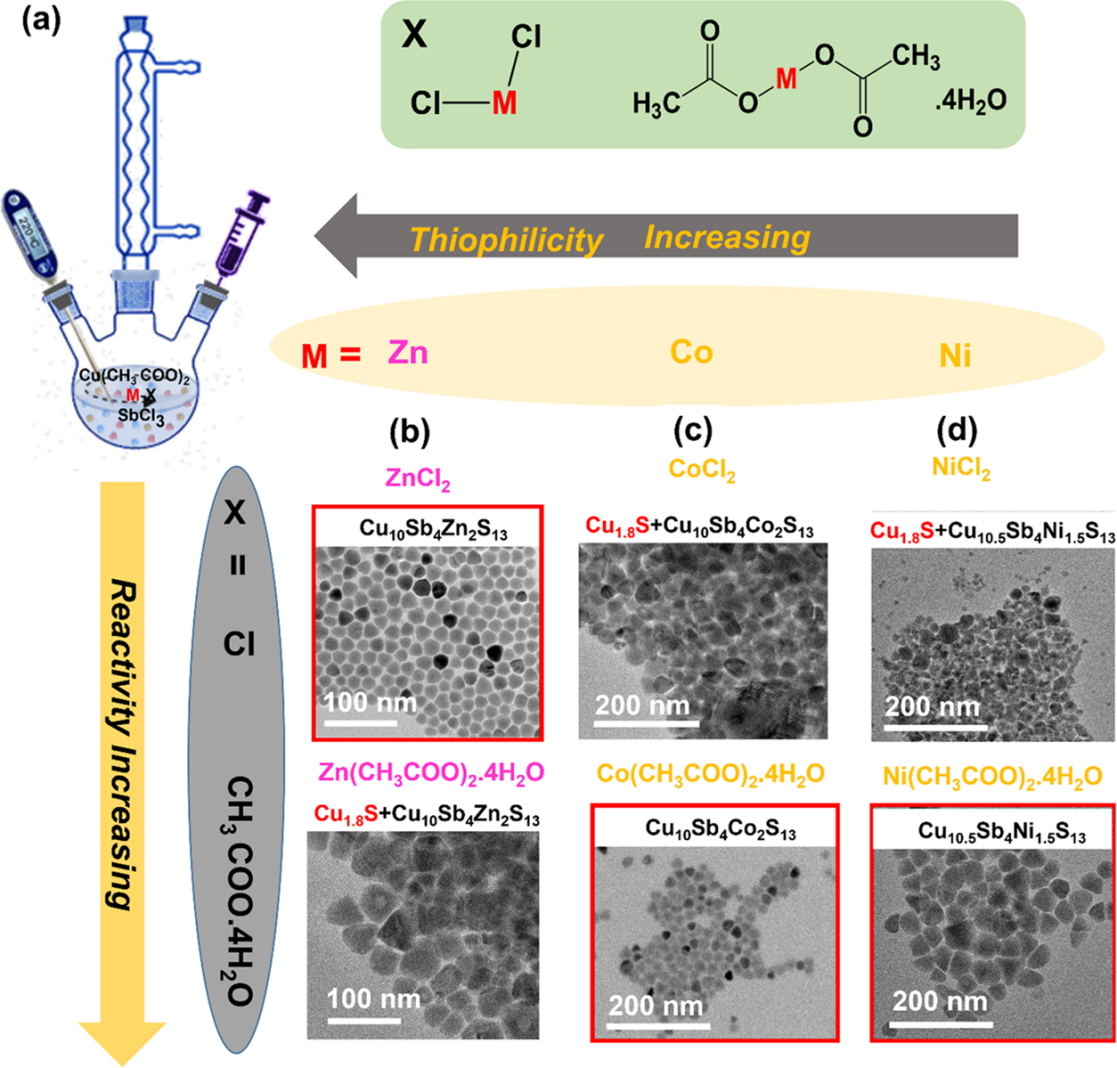
(a) Schematic for the synthesis of the tetrahedrite-substituted NCs, and TEM images of the NCs synthesized with different precursors of (b) Zn, (c) Co, and (d) Ni.

The findings compiled in Figure 5 demonstrate that phase control synthesis of tetrahedrite-substituted chalcogenides is attainable through a colloidal hot injection approach; however, it involves the suitable balancing of precursor reactivities. The hard and soft acid–base (HSAB) theory serves as a useful guide for predicting the reactivity of metal precursors. Based on the thiophilicity trends, Ni, Co, and Zn tend to form sulfur bonds more slowly compared to Cu. Therefore, Cu(CH_3_COO)_2_, a Cu precursor of intermediate reactivity, was selected for combination with SbCl_3_. A series of experiments were conducted to investigate the reactivity of different transition metal (Ni, Co, and Zn) precursors to get pure-phase-substituted tetrahedrite NCs (Figure S10). The Cu_10_Sb_4_Zn_2_S_13_ NCs were synthesized by combining Zn in halide form, Cu in acetate, and Sb in halide form with thiol as an S source, while for the synthesis of Cu_10_Co_2_Sb_4_S_13_ and Cu_10.5_Ni_1.5_Sb_4_S_13_ NCs, hydrated acetates of Ni and Co were optimal. Strong interactions between chloride ions and Zn-ions make ZnCl_2_ less reactive compared to Zn(CH_3_COO)_2_·4H_2_O. Our observations show that in the presence of chloride, the overall rate of reaction between Cu precursors should be increased, while there was a decrease in the rate of reaction between the Zn precursor and S. This results in pure phase Cu_10_Sb_4_Zn_2_S_13_ NCs. In contrast, the use of the chloride-based Ni precursor (NiCl_2_) results in the growth of Cu*_x_*S byproducts (Figure S10). It is known that Ni^2+^ and Co^2+^ are harder Lewis acids than Zn^2+^.^42^ Therefore, to cope with the Cu precursor of intermediate reactivity, in the case of Cu_10_Co_2_Sb_4_S_13_ and Cu_10.5_Ni_1.5_Sb_4_S_13_, the use of more reactive Ni and Co precursors Co(CH_3_COO)_2_·4H_2_O and Ni(CH_3_COO)_2_·4H_2_O, respectively, yielded pure phase Cu_10_Sb_4_Co_2_S_13_ and Cu_10.5_Sb_4_Ni_1.5_S_13_ NCs.

Mechanistically, the formation of multinary NCs can take place either directly from the solution containing a mixture of precursors or through a solid-state reaction.^7,39^ According to the HSAB theory, the metal precursors will react with the S precursor (a soft Lewis base) in the order of Cu^2+^ > Zn^2+^ > Sb^3+^. However, the presence of coordinating ligands in the reaction mixture and their different coordination with different metal precursors adds an extra layer of complexity.^43^ The reported literature based on mechanistic insight for the synthesis of the ternary Cu–Sb–S system depicts that the in situ formed Cu_x_S seed is responsible for the growth of Cu–Sb–S NCs.^44^ For all three substituted tetrahedrite NCs (Cu_10_Zn_2_Sb_4_S_13_, Cu_10_Co_2_Sb_4_S_13_, and Cu_10.5_Ni_1.5_Sb_4_S_13_), Cu*_x_*S was observed at the initial stages, suggesting that the formation of quaternary NCs followed a successive cation-exchange mechanism between Cu^2+^ and foreign cations (Sb^3+^ and Zn^2+^). Based on the observations discussed in the previous section (Figure 4) and the results described above (Figure 5), we can relate the growth mechanism with the reactivity of transition metal precursors. In the case of Cu_10_Zn_2_Sb_4_S_13_, after the formation of (Cu^+^)_10_(Cu^2+^)_2_Sb_4_S_13_, the less reactive chloride-based Zn precursor results in unequal diffusion on the same S^2–^ anion frameworks between Cu and Zn, resulting in the formation of hollow particles for an aliquot taken after 10 min, which further transformed to solid Cu_10_Zn_2_Sb_4_S_13_ NCs after 15 min of reaction with complete cation exchange between Cu^2+^ and Zn^2+^. On the other hand, for Cu_10_Co_2_Sb_4_S_13_ and Cu_10.5_Ni_1.5_Sb_4_S_13_, the diffusion of Ni and Co with more reactive acetate-based Co and Ni precursors is so fast that it results in quaternary composition within 5 min of reaction. These findings suggest that the metal-to-ligand complex that formed in the case of Cu_10_Co_2_Sb_4_S_13_ and Cu_10.5_Ni_1.5_Sb_4_ S_13_ is not strong compared to Cu_10_Zn_2_Sb_4_S_13_, which results in the evolution of cubic phase NCs after 5 min because of faster ionic diffusion of Ni and Co compared to Zn for Cu_10_Zn_2_Sb_4_S_13_.

## 5 Transport Properties

**Figure 6:**
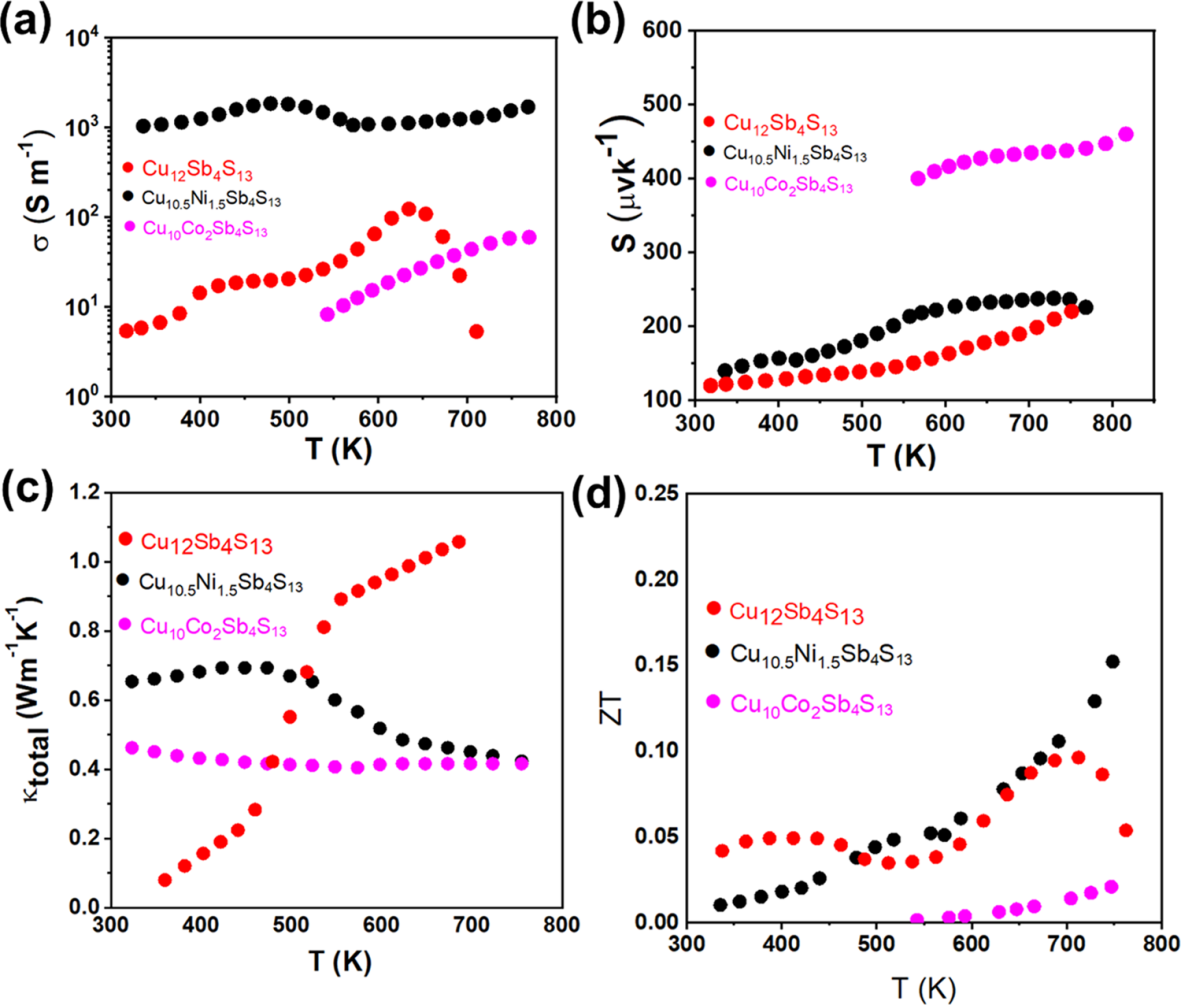
Temperature-dependent (a) electrical conductivity, σ, (b) Seebeck coefficient, *S*, (c) total thermal conductivity, κ_total_, and (d) thermoelectric figure of merit, *ZT*, values for Cu_10_Sb_4_S_13_, Cu_10_Co_2_Sb_4_S_13_, and Cu_10.5_Ni_1.5_Sb_4_S_13_.

The transport properties of pure (Cu_12_Sb_4_S_13_) and substituted tetrahedrites (Cu_10_Zn_2_Sb_4_S_13_, Cu_10_Co_2_Sb_4_S_13_, and Cu_10.5_Sb_4_Ni_1.5_S_13_) were investigated in the temperature range of 300–700 K. Figure 6 shows the electrical conductivity (σ), Seebeck coefficient (*S*), thermal conductivity (κ), and overall thermoelectric figure of merit (*ZT* = *S*^2^σ*T*/κ) for Cu_12_Sb_4_S_13_, Cu_10_Co_2_Sb_4_S_13_, and Cu_10.5_Ni_1.5_Sb_4_S_13_ NCs. The transport properties show an increase in electrical conductivity and power factor with a decrease in thermal conductivity upon substitution of the transition metal (Co and Ni) (Figure 6). While all of the synthesized crystal phases display outstanding low thermal conductivity, the Cu_10.5_Sb_4_Ni_1.5_S_13_ system shows the most enhanced electrical conductivity compared to Cu_10_Zn_2_Sb_4_S_13_ and Cu_10_Co_2_Sb_4_S_13_.^45^ The optical bandgaps were reduced relative to the pure tetrahedrite for the synthesized material (Figures S15d and S16 and Table S2).

The hybridization of Cu 3d and S 3p orbitals results in the formation of pure tetrahedrite (Cu^+^)_10_(Cu^2+^)_2_Sb_4_S_13_. The valence bands of S and Sb p orbitals are separated from the conduction bands in pure tetrahedrite by a clearly defined energy gap. The tetrahedrite material generally behaves as a degenerated p-type semiconductor. This heavy p-type doping is associated with the existence of an extra S^2–^ ion and two unfilled holes, which is maintained by covalent interactions with the s and d orbitals of neighboring Cu^+^ ions on the 12e sites. The electric resistivity of pellets by substituting Cu with the d^10^ cation (Zn^2+^) is higher (Table S2). Previous reports and DFT calculations have demonstrated that two Zn atoms are substituted for Cu, and as a result, the holes in the valence band are filled with the extra 4s electrons, and the material turns insulating.^45^ Similar behavior is consistent with the results observed here for Cu_12–*x*_Zn*_x_*Sb_4_S_13_ NCs with *x* = 2 in the tetrahedrite structure (Cu_12_Sb_4_S_13_), suggesting that the material is an insulator. In contrast, Ni-containing materials show the highest electrical conductivity and lowest Seebeck coefficient, which clearly indicates a large increase in the charge carrier concentration. The electrical conductivity of Cu_10.5_Ni_1.5_Sb_4_S_13_ NCs (Figure 6a) does not show a clear evolution with temperature as two effects counteract the thermal excitation of the additional charge carrier and their lattice scattering with increasing temperature. The thermal excitation of electrons in the impurity state from the valence band is what causes the slight reduction in electrical conductivity above 400 °C. Ni-doping due to the hybridization of Cu 3d states and S 3p states with the valence band of Ni 3d states produces spin splitting, which results in higher electrical conductivity of the Ni-substituted NCs. Thus, thermal excitation of the carriers becomes easier as a result of a narrower bandgap.^37^ The optical data and Tauc plot of the as-synthesized Cu_10.5_Ni_1.5_Sb_4_S_13_ NCs are provided in Figure S16c,d.

The Seebeck coefficient (*S*) for Cu_10_Sb_4_S_13_, Cu_10_Co_2_Sb_4_S_13_, and Cu_10.5_Ni_1.5_Sb_4_S_13_ NCs (Figure 6b) is positive over the whole temperature range, indicating a p-type conductivity where holes make up the majority of the charge carriers. The mean heat capacity (*C*_p_) used in this study to calculate the thermal conductivity was 0.45 J g^–1^ K^–1^.^46^ The synthesized materials display remarkably low thermal conductivity (Figure 6c), well below 1 W m^–1^ K^–1^, which is associated with the crystal structure, complex composition, and the large density of grain boundaries. The decrease in the thermal conductivity of Cu_10_Co_2_Sb_4_S_13_ and Cu_10.5_Ni_1.5_Sb_4_S_13_ with the increase in temperature can be explained on the basis of phonon scattering enhancement.^46^ Overall, the *ZT* value (Figure 6d) of all substituted NCs increases with the increase in temperature. The transport properties for tetrahedrite-based samples vary widely depending on factors such as the synthesis and sintering conditions, dopant, and doping levels.^28^ Thus, the NCs synthesized using the hot injection reaction protocol with different dopants (Zn, Ni, and Co) show different thermoelectric properties depending on the level of the dopant (*x* = 1.5–2), highlighting the role of the dopant and doping level on the thermoelectric figures.

## 6 Conclusions

In summary, we have shown that tetrahedrite-substituted NCs can be formed by a hot injection process that requires less energy and time compared to traditional solid-state methods. This research focused on a generalized synthesis approach for pure-phase pyramid-shaped NCs by optimizing the reactivity of precursors with different reaction parameters. The evolution of pure phases for the bottom-up production of substituted tetrahedrite has been identified by examining the progress of the reactions over time. The key intermediate identified within the growth process is Cu_1.8_S and is observed consistently for all of the three substituted NCs. The synthesis approach is simple, yielding exclusive quaternary NCs of controlled shape and size, and is sufficiently versatile to be exploited for the future synthesis of a large variety of I–II–V–VI-based NCs. The transport properties of these samples showed that the Ni-substituted NCs with Cu_10.5_Sb_4_Ni_1.5_S_13_ composition exhibit the most promising transport properties compared to that of Zn or Co. These findings suggest that by optimizing the level of substitution of the transition metal in the crystal structure of tetrahedrite (Cu_12–*x*_Sb_4_S_13_), the thermoelectric performance of these NCs can be further tuned.

## Supporting Information Available

Additional data of size distribution histograms, Rietveld refinement, Raman, and XPS, along with UV–vis spectra of the synthesized nanocrystals (PDF)

## References

[ref1] Coughlan C.; Ibanez M.; Dobrozhan O.; Singh A.; Cabot A.; Ryan K. M. Compound copper chalcogenide nanocrystals. Chem. Rev. 2017, 117, 5865–6109. 10.1021/acs.chemrev.6b00376. 28394585

[ref2] Gao M.-R.; Xu Y.-F.; Jiang J.; Yu S.-H. Nanostructured metal chalcogenides: synthesis, modification, and applications in energy conversion and storage devices. Chem. Soc. Rev. 2013, 42, 2986–3017. 10.1039/c2cs35310e. 23296312

[ref3] Guijarro N.; Prévot M. S.; Yu X.; Jeanbourquin X. A.; Bornoz P.; Bourée W.; Johnson M.; Le Formal F.; Sivula K. A bottom-up approach toward all-solution-processed high-efficiency Cu (In, Ga) S2 photocathodes for solar water splitting. Adv. Energy Mater. 2016, 6, 1501949 10.1002/aenm.201501949.

[ref4] Panthani M. G.; Akhavan V.; Goodfellow B.; Schmidtke J. P.; Dunn L.; Dodabalapur A.; Barbara P. F.; Korgel B. A. Synthesis of CuInS2, CuInSe2, and Cu (In x Ga1-x) Se2 (CIGS) nanocrystal “inks” for printable photovoltaics. J. Am. Chem. Soc. 2008, 130, 16770–16777. 10.1021/ja805845q. 19049468

[ref5] Singh S.; Ryan K. M. Occurrence of polytypism in compound colloidal metal chalcogenide nanocrystals, opportunities, and challenges. J. Phys. Chem. Lett. 2015, 6, 3141–3148. 10.1021/acs.jpclett.5b01311.

[ref6] Stroyuk O.; Raevskaya A.; Gaponik N. Solar light harvesting with multinary metal chalcogenide nanocrystals. Chem. Soc. Rev. 2018, 47, 5354–5422. 10.1039/C8CS00029H. 29799031

[ref7] Xia C.; Pedrazo-Tardajos A.; Wang D.; Meeldijk J. D.; Gerritsen H. C.; Bals S.; de Mello Donega C. Seeded Growth Combined with Cation Exchange for the Synthesis of Anisotropic Cu2–x S/ZnS, Cu2–x S, and CuInS2 Nanorods. Chem. Mater. 2021, 33, 102–116. 10.1021/acs.chemmater.0c02817. 33456135 PMC7808334

[ref8] Ning J.; Duan Z.; Kershaw S. V.; Rogach A. L. Phase-Controlled Growth of CuInS2 Shells to Realize Colloidal CuInSe2/CuInS2 Core/Shell Nanostructures. ACS Nano 2020, 14, 11799–11808. 10.1021/acsnano.0c04660. 32865971

[ref9] Ren H.; Li Z.; Sun Y.; Gao P.; McCarthy C.; Liu N.; Xu H.; Ryan K. M. Precursor-Mediated Linear-and Branched-Polytypism Control in CuαZnβSnγSeδ Colloidal Nanocrystals Using a Dual-Injection Method. Chem. Mater. 2020, 32, 7254–7262. 10.1021/acs.chemmater.0c01663.

[ref10] Xia C.; Winckelmans N.; Prins P. T.; Bals S.; Gerritsen H. C.; de Mello Donega C. Near-infrared-emitting CuInS2/ZnS dot-in-rod colloidal heteronanorods by seeded growth. J. Am. Chem. Soc. 2018, 140, 5755–5763. 10.1021/jacs.8b01412. 29569443 PMC5934729

[ref11] Pundsack T. J.; Chernomordik B. D.; Béland A. E.; Aydil E. S.; Blank D. A. Excited-state dynamics in CZTS nanocrystals. J. Phys. Chem. Lett. 2013, 4, 2711–2714. 10.1021/jz4013245.

[ref12] Singh S.; Brandon M.; Liu P.; Laffir F.; Redington W.; Ryan K. M. Selective phase transformation of wurtzite Cu2ZnSn (SSe) 4 (CZTSSe) nanocrystals into zinc-blende and kesterite phases by solution and solid state transformations. Chem. Mater. 2016, 28, 5055–5062. 10.1021/acs.chemmater.6b01845.

[ref13] Tang J.; Hinds S.; Kelley S. O.; Sargent E. H. Synthesis of Colloidal CuGaSe2, CuInSe2, and Cu (InGa) Se2 Nanoparticles. Chem. Mater. 2008, 20, 6906–6910. 10.1021/cm801655w.

[ref14] Wu L.; Wang Q.; Zhuang T.-T.; Li Y.; Zhang G.; Liu G.-Q.; Fan F.-J.; Shi L.; Yu S.-H. Single crystalline quaternary sulfide nanobelts for efficient solar-to-hydrogen conversion. Nat. Commun. 2020, 11, 5194 10.1038/s41467-020-18679-z. 33060575 PMC7567062

[ref15] Kapuria N.; Ghorpade U. V.; Zubair M.; Mishra M.; Singh S.; Ryan K. M. Metal chalcogenide semiconductor nanocrystals synthesized from ion-conducting seeds and their applications. J. Mater. Chem. C 2020, 8, 13868–13895. 10.1039/D0TC02895A.

[ref16] Coughlan C.; Guo Y.; Singh S.; Nakahara S.; Ryan K. M. Synthesis of Curved CuIn1–x Ga x (S1–y Se y) 2 Nanocrystals and Complete Characterization of Their Diffraction Contrast Effects. Chem. Mater. 2018, 30, 8679–8689. 10.1021/acs.chemmater.8b04082.

[ref17] Singh S.; Liu P.; Singh A.; Coughlan C.; Wang J.; Lusi M.; Ryan K. M. Colloidal Cu2ZnSn (SSe) 4 (CZTSSe) nanocrystals: shape and crystal phase control to form dots, arrows, ellipsoids, and rods. Chem. Mater. 2015, 27, 4742–4748. 10.1021/acs.chemmater.5b01399.

[ref18] Wang Y.-H. A.; Zhang X.; Bao N.; Lin B.; Gupta A. Synthesis of shape-controlled monodisperse wurtzite CuIn x Ga1–x S2 semiconductor nanocrystals with tunable band gap. J. Am. Chem. Soc. 2011, 133, 11072–11075. 10.1021/ja203933e. 21702462

[ref19] Fan F.-J.; Yu B.; Wang Y.-X.; Zhu Y.-L.; Liu X.-J.; Yu S.-H.; Ren Z. Colloidal synthesis of Cu2CdSnSe4 nanocrystals and hot-pressing to enhance the thermoelectric figure-of-merit. J. Am. Chem. Soc. 2011, 133, 15910–15913. 10.1021/ja207159j. 21910492

[ref20] Ibáñez M.; Zamani R.; LaLonde A.; Cadavid D.; Li W.; Shavel A.; Arbiol J.; Morante J. R.; Gorsse S.; Snyder G. J.; Cabot A. Cu2ZnGeSe4 Nanocrystals: Synthesis and Thermoelectric Properties. J. Am. Chem. Soc. 2012, 134, 4060–4063. 10.1021/ja211952z. 22332903

[ref21] Singh A.; Geaney H.; Laffir F.; Ryan K. M. Colloidal Synthesis of Wurtzite Cu2ZnSnS4 Nanorods and Their Perpendicular Assembly. J. Am. Chem. Soc. 2012, 134, 2910–2913. 10.1021/ja2112146. 22296030

[ref22] Yarema O.; Yarema M.; Moser A.; Enger O.; Wood V. Composition-and size-controlled I–V–VI Semiconductor nanocrystals. Chem. Mater. 2020, 32, 2078–2085. 10.1021/acs.chemmater.9b05191.

[ref23] Bera S.; Shyamal S.; Sen S.; Pradhan N. Insights of Crystal Growth, Nucleation Density, and Shape Modulations in the Formation of I–V–VI Ternary Semiconductor Nanoplatelet Photoelectrocatalysts. J. Phys. Chem. C 2020, 124, 15607–15615. 10.1021/acs.jpcc.0c03947.

[ref24] Yang B.; Wang L.; Han J.; Zhou Y.; Song H.; Chen S.; Zhong J.; Lv L.; Niu D.; Tang J. CuSbS2 as a promising earth-abundant photovoltaic absorber material: a combined theoretical and experimental study. Chem. Mater. 2014, 26, 3135–3143. 10.1021/cm500516v.

[ref25] Lu X.; Morelli D. T.; Wang Y.; Lai W.; Xia Y.; Ozolins V. Phase Stability, Crystal Structure, and Thermoelectric Properties of Cu12Sb4S13–x Se x Solid Solutions. Chem. Mater. 2016, 28, 1781–1786. 10.1021/acs.chemmater.5b04796.

[ref26] Bera S.; Dutta A.; Mutyala S.; Ghosh D.; Pradhan N. Predominated thermodynamically controlled reactions for suppressing cross nucleations in formation of multinary substituted tetrahedrite nanocrystals. J. Phys. Chem. Lett. 2018, 9, 1907–1912. 10.1021/acs.jpclett.8b00680. 29584942

[ref27] Alqahtani T.; Khan M. D.; Lewis D. J.; Zhong X. L.; O’Brien P. Scalable synthesis of Cu–Sb–S phases from reactive melts of metal xanthates and effect of cationic manipulation on structural and optical properties. Sci. Rep. 2021, 11, 1887 10.1038/s41598-020-80951-5. 33479247 PMC7820284

[ref28] Nakada T.; Takahashi M.; Shijimaya C.; Higashimine K.; Zhou W.; Dwivedi P.; Ohta M.; Takida H.; Akatsuka T.; Miyata M.; Maenosono S. Gram-scale synthesis of tetrahedrite nanoparticles and their thermoelectric properties. Langmuir 2019, 35, 16335–16340. 10.1021/acs.langmuir.9b03003. 31715104

[ref29] Barbier T.; Lemoine P.; Gascoin S.; Lebedev O. I.; Kaltzoglou A.; Vaqueiro P.; Powell A. V.; Smith R. I.; Guilmeau E. Structural stability of the synthetic thermoelectric ternary and nickel-substituted tetrahedrite phases. J. Alloys Compd. 2015, 634, 253–262. 10.1016/j.jallcom.2015.02.045.

[ref30] Suekuni K.; Tsuruta K.; Ariga T.; Koyano M. Thermoelectric properties of mineral tetrahedrites Cu10Tr2Sb4S13 with low thermal conductivity. Appl. Phys. Express 2012, 5, 051201 10.1143/APEX.5.051201.

[ref31] Weller D. P.; Stevens D. L.; Kunkel G. E.; Ochs A. M.; Holder C. F.; Morelli D. T.; Anderson M. E. Thermoelectric performance of tetrahedrite synthesized by a modified polyol process. Chem. Mater. 2017, 29, 1656–1664. 10.1021/acs.chemmater.6b04950.

[ref32] Anderson M. E.; Bharadwaya S.; Schaak R. Modified polyol synthesis of bulk-scale nanostructured bismuth antimony telluride. J. Mater. Chem. 2010, 20, 8362–8367. 10.1039/c0jm01424a.

[ref33] Weller D.; Kunkel G.; Ochs A.; Morelli D.; Anderson M. E. Observation of n-type behavior in Fe-doped tetrahedrite at low temperature. Mater. Today Phys. 2018, 7, 1–6. 10.1016/j.mtphys.2018.10.003.

[ref34] Fasana C. D.; Jensen M. S.; Ponte G. E. G.; MacAlister T. R.; Kunkel G. E.; Rogers J. P.; Ochs A. M.; Stevens D. L.; Weller D. P.; Morelli D. T. Synthetic versatility, reaction pathway, and thermal stability of tetrahedrite nanoparticles. J. Mater. Chem. C 2020, 8, 14219–14229. 10.1039/D0TC03599H.

[ref35] Inada Y.; Ozutsumi K.; Funahashi S.; Soyama S.; Kawashima T.; Tanaka M. Structure of copper (II) ethylenediamine complexes in aqueous and neat ethylenediamine solutions and solvent-exchange kinetics of the copper (II) ion in ethylenediamine as studied by EXAFS and NMR methods. Inorg. Chem. 1993, 32, 3010–3014. 10.1021/ic00066a009.

[ref36] Xie Y.; Riedinger A.; Prato M.; Casu A.; Genovese A.; Guardia P.; Sottini S.; Sangregorio C.; Miszta K.; Ghosh S.; et al. Copper sulfide nanocrystals with tunable composition by reduction of covellite nanocrystals with Cu+ ions. J. Am. Chem. Soc. 2013, 135, 17630–17637. 10.1021/ja409754v. 24128337

[ref37] Sarilmaz A.; Ozel F. Synthesis of band-gap tunable earth-abundant CXTS (X = Mn+ 2, Co+ 2, Ni+ 2 and Zn+ 2) nanorods: Toward a generalized synthesis strategy of quaternary chalcogenides. J. Alloys Compd. 2019, 780, 518–522. 10.1016/j.jallcom.2018.11.370.

[ref38] Sarswat P. K.; Free M. L. Enhanced photoelectrochemical response from copper antimony zinc sulfide thin films on transparent conducting electrode. Int. J. Photoenergy 2013, 2013, 154694 10.1155/2013/154694.

[ref39] Liang Q.; Huang K.; Ren X.; Zhang W.; Xie R.; Feng S. Synthesis of Cu–Sb–S nanocrystals: insight into the mechanism of composition and crystal phase selection. CrystEngComm 2016, 18, 3703–3710. 10.1039/C6CE00474A.

[ref40] Lox J. F. L.; Dang Z.; Dzhagan V. M.; Spittel D.; Martín-García B.; Moreels I.; Zahn D. R.; Lesnyak V. Near-infrared Cu–In–Se-based colloidal nanocrystals via cation exchange. Chem. Mater. 2018, 30, 2607–2617. 10.1021/acs.chemmater.7b05187.

[ref41] Lox J. F. L.; Dang Z.; Lê Anh M.; Hollinger E.; Lesnyak V. Colloidal Cu–Zn–In–S-Based Disk-Shaped Nanocookies. Chem. Mater. 2019, 31, 2873–2883. 10.1021/acs.chemmater.9b00005.

[ref42] Mantella V.; Varandili S. B.; Pankhurst J. R.; Buonsanti R. Colloidal synthesis of Cu–M–S (M = V, Cr, Mn) nanocrystals by tuning the copper precursor reactivity. Chem. Mater. 2020, 32, 9780–9786. 10.1021/acs.chemmater.0c03788.

[ref43] Xie R.; Rutherford M.; Peng X. Formation of high-quality I– III– VI semiconductor nanocrystals by tuning relative reactivity of cationic precursors. J. Am. Chem. Soc. 2009, 131, 5691–5697. 10.1021/ja9005767. 19331353

[ref44] Just J.; Coughlan C.; Singh S.; Ren H.; Müller O.; Becker P.; Unold T.; Ryan K. M. Insights into Nucleation and Growth of Colloidal Quaternary Nanocrystals by Multimodal X-ray Analysis. ACS Nano 2021, 15, 6439–6447. 10.1021/acsnano.0c08617. 33770436 PMC8291568

[ref45] Lu X.; Morelli D. T.; Xia Y.; Ozolins V. Increasing the thermoelectric figure of merit of tetrahedrites by co-doping with nickel and zinc. Chem. Mater. 2015, 27, 408–413. 10.1021/cm502570b.

[ref46] Heo J.; Laurita G.; Muir S.; Subramanian M. A.; Keszler D. A. Enhanced thermoelectric performance of synthetic tetrahedrites. Chem. Mater. 2014, 26, 2047–2051. 10.1021/cm404026k.

