# Supplementary material for: Precursor-Mediated Colloidal Synthesis of Compositionally Tunable Cu–Sb–M–S (M = Zn, Co, and Ni) Nanocrystals and Their Transport Properties
Source: Chem Mater. 2022 Nov 21;34(23):10528–37. doi: 10.1021/acs.chemmater.2c02605 (PMC9753559; doi:10.1021/acs.chemmater.2c02605)
Supplement: Supplementary file 1 — cm2c02605_si_001.pdf [file cm2c02605_si_001.pdf]

Supporting Information

Precursor Mediated Colloidal Synthesis of Compositionally Tunable Cu-Sb-M-S (M= Zn, Co and Ni) Nanocrystals and their Transport Properties.

Maria Zubair,‡ Vasily A Lebedev,‡ Mohini Mishra,‡ Temilade Esther Adegoke,‡ , Ibrahim Saana Amiinu,‡ Yu Zhang § Andreu Cabot,§¥ Shalini Singh,‡ Kevin M. Ryan‡*

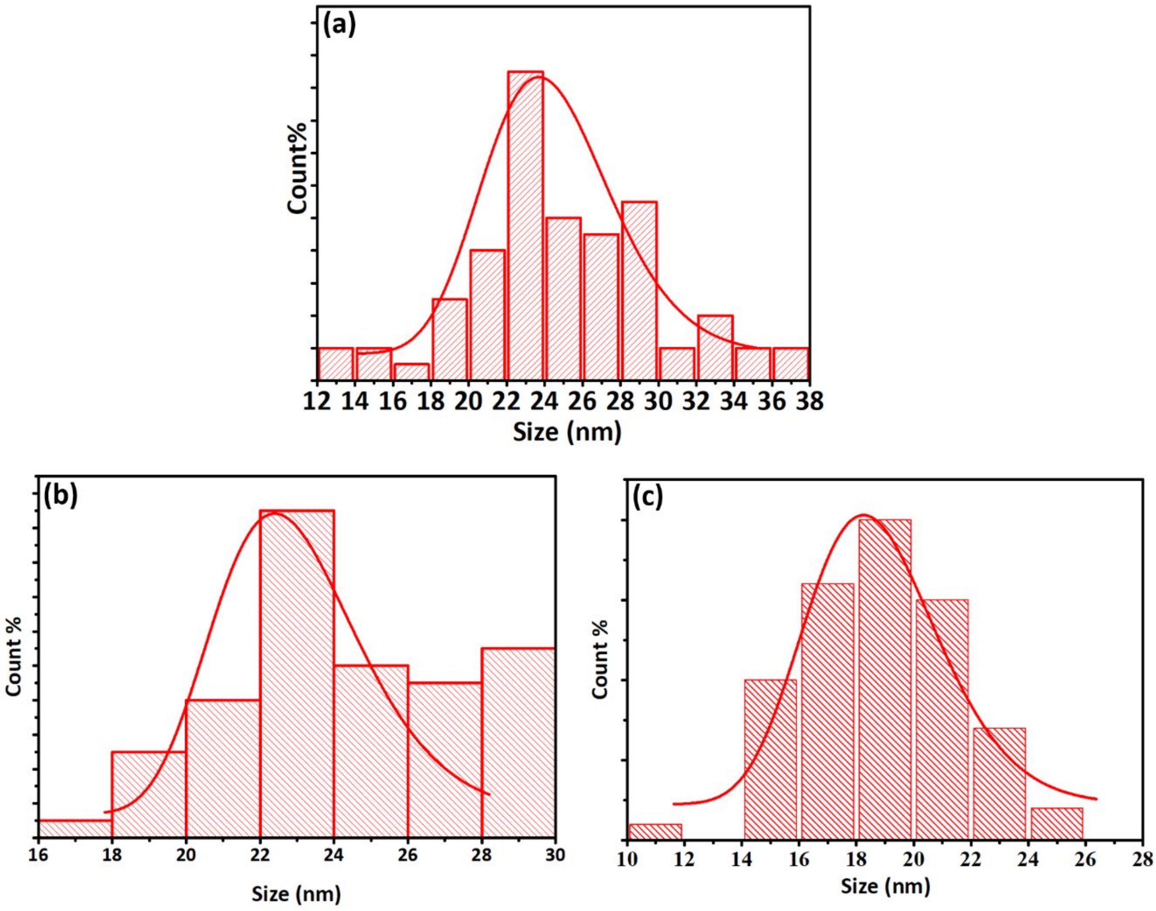

Figure S1. Size distribution histogram of NCs (a) $Cu_{10}Zn_2Sb_4S_{13}$ (b) $Cu_{10.5}Sb_4Ni_{1.5}S_{13}$ and (c) $Cu_{10}Sb_4Co_2S_{13}$. The average diameters were obtained by analysing at least 100 particles in each case.

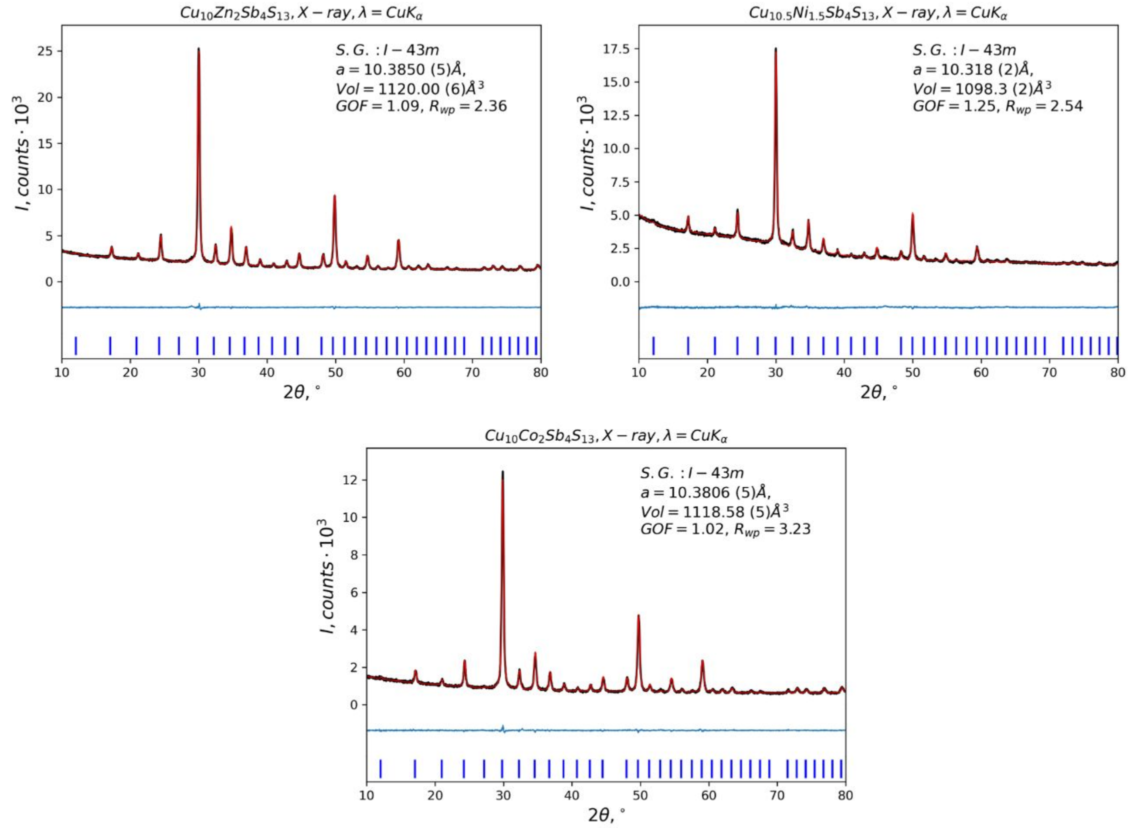

Figure S2: Rietveld refinement graphs of the XRD pattern of $Cu_{10}Zn_2Sb_4S_{13}$, $Cu_{10.5}Ni_{1.5}Sb_4S_{13}$, and $Cu_{10}Co_2Sb_4S_{13}$.

According to the phase analysis, a single crystalline phase has been observed in each of samples. These phases can be described as isostructural to the tetrahedrite (#04-021-9982 card (PDF-4+), $Cu_{10.5}Sb_4Ni_{1.5}S_{13}$, S.G. I-43m , a = 10.363(6)). Rietveld refinements were performed using Jana 2006 software [Petricek, V., Dusek, M. & Palatinus, L. (2014). Z. Kristallogr. 229(5), 345-352. DOI 10.1515/zkri-2014-1737]. The background function was chosen as a Legendre polynomial of 9th order with 1/x term. The peak profile was described as pseudo-Voight

functions. The CIF file of #04-021-9982 card was used as a source of initial atomic coordinates. Lattice parameters were refined for each of the substituted structures.

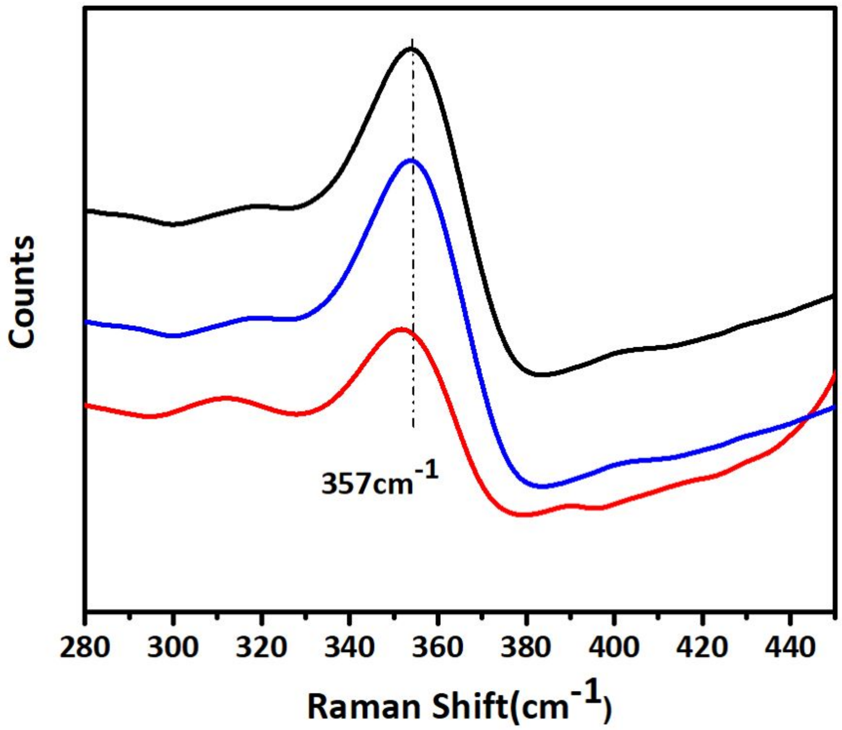

Figure S3: (a) Raman spectra of $Cu_{10}Sb_4Zn_2S_{13}$ (black) $Cu_{10}Sb_4Co_2S_{13}$ (red) and $Cu_{10.5}Sb_4Ni_{1.5}S_{13}$ (blue) NCs.

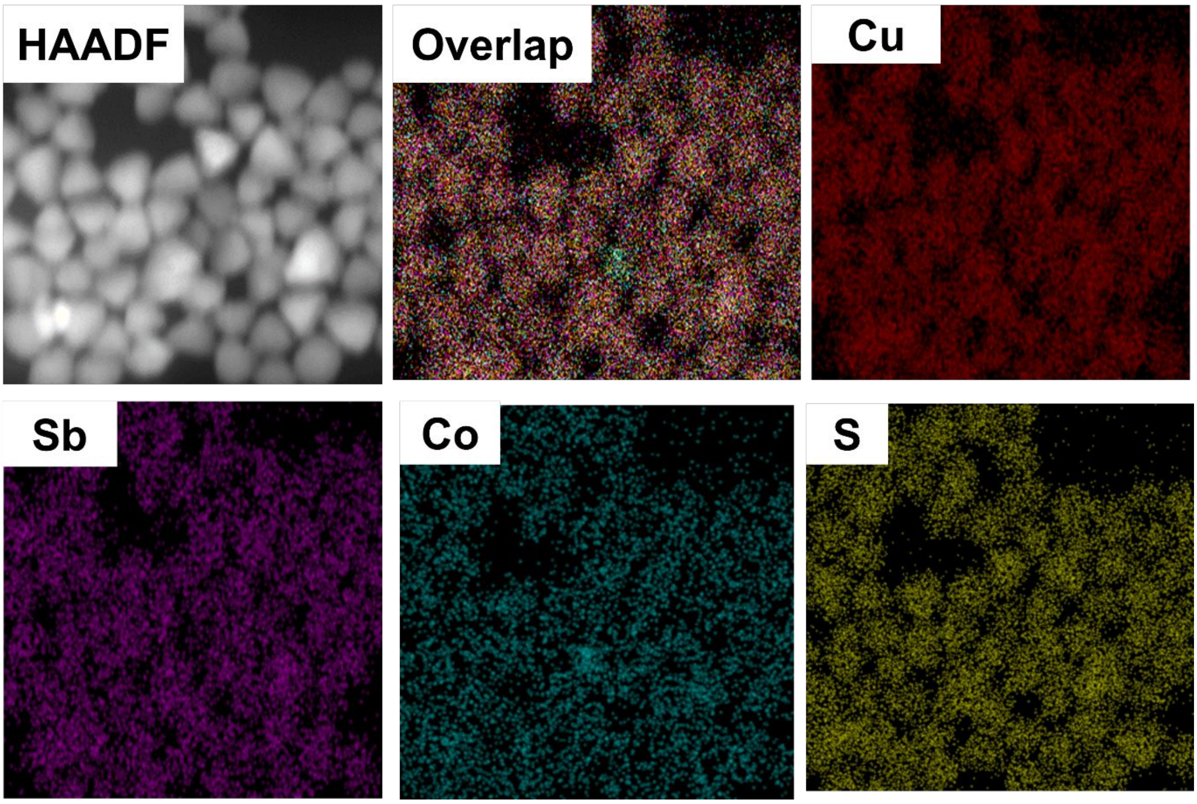

Figure S4: HAADF-STEM image of a $Cu_{10}Sb_4Co_2S_{13}$ NCs with corresponding STEM-EDS elemental maps related to Cu (red), Zn (green), Sb (purple), and S (yellow) of $Cu_{10}Sb_4Co_2S_{13}$ NCs.

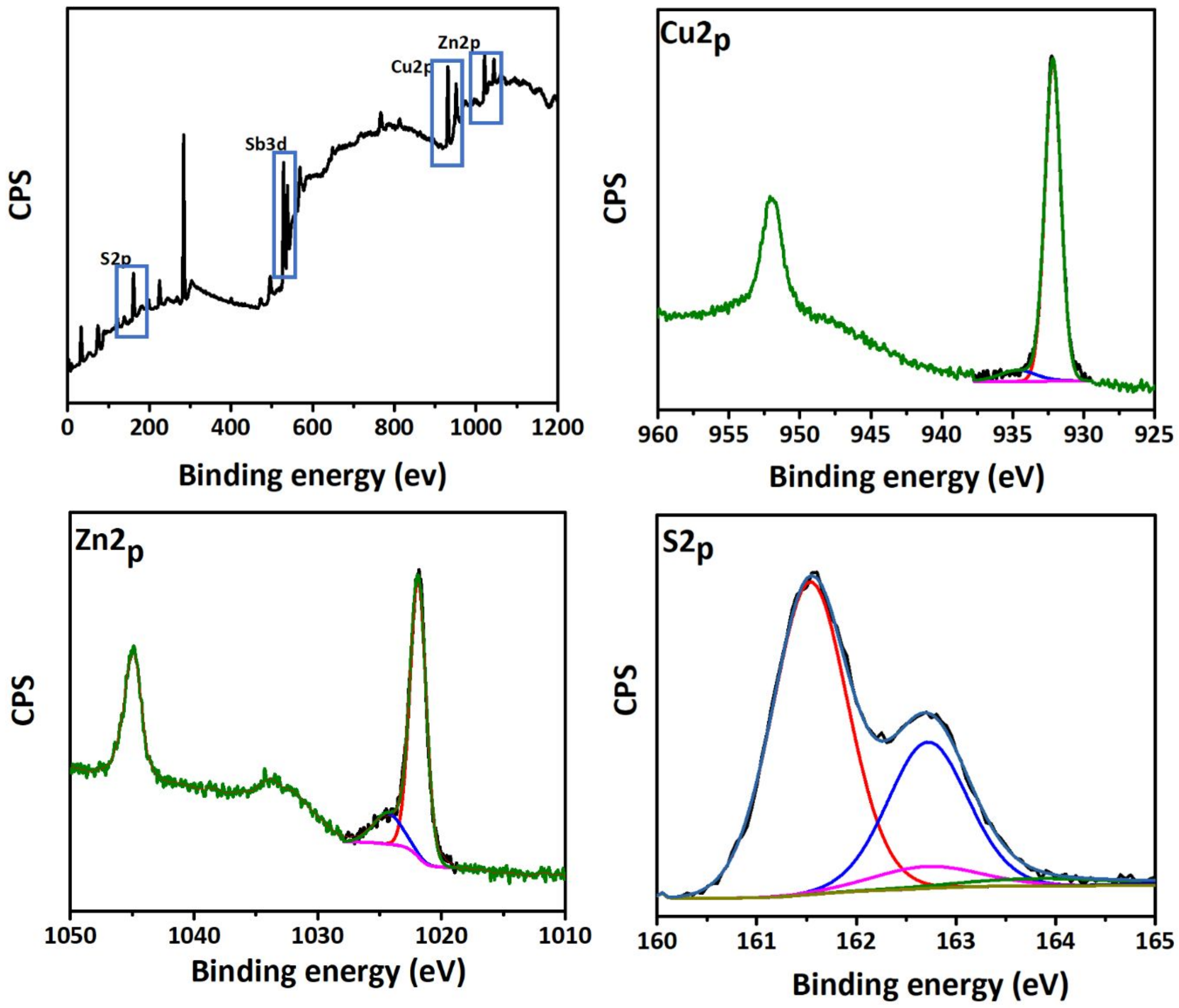

Figure S5: b) XPS spectra of $Cu_{10.5}Sb_4Zn_2S_{13}$ NCs with (c) XPS of Cu (2p), (d) XPS of S (2p), XPS of Sb (3d) and XPS of Zn (2p) for $Cu_{12-x}Sb_4Zn_xS_{13}$.

XPS survey spectrum of $Cu_{10}Sb_4Zn_2S_{13}$ NCs is presented in Figure S4. It shows the presence of Cu, Zn, Sn, Se, S, O, C, and N. The ligands that cap the nanocrystals and the solvent (toluene) used to disperse them cause the presence of N, C, and O. In the Cu XPS spectrum, an intense narrow set of doublet peaks positioned at 951.52 eV (2p1/2) and 931.75 eV (2p3/2) was observed. The presence of Cu in a Cu^{+1} state was confirmed by the two peaks, which were observed at a difference of 19.77 eV. However, doublet peaks observed at 1044.63 eV (2p1/2) and 1021.46 eV (2p3/2) of Zn 2p confirmed the presence of Zn^{2+}.

Table S1: ICP data of $Cu_{10}Zn_2Sb_4S_{13}$, $Cu_{10}Co_2Sb_4S_{13}$ and $Cu_{10.5}Ni_{1.5}Sb_4S_{13}$ NCs.

CAZS	Cu		Zn		Sb	
	Wt	atoms	Wt	atoms	Wt	atoms
C1	28.23		7.83		17.47	
C2	28.08		7.49		17.52	
C3	28.27		7.54		17.68	
avg	28.19	0.443	7.62	0.11	17.55	0.14
	ppm					
**CANS**	**Cu**		**Co**		**Sb**	
	Wt	atoms	Wt	atoms	Wt	atoms
C1	26.41		8.93		17.42	
C2	26.53		8.98		17.46	
C3	26.65		9.01		17.85	
avg	26.53	0.417	8.97	0.15	17.57	0.14
	ppm					
**CACS**	**Cu**		**Ni**		**Sb**	
	Wt	atoms	Wt	atoms	Wt	atoms
C1	31.35		4.88		18.63	
C2	31.2		4.92		18.03	
C3	31.15		4.85		18.97	
avg	31.2	0.49	4.88	0.08	18.5	0.15
	ppm					

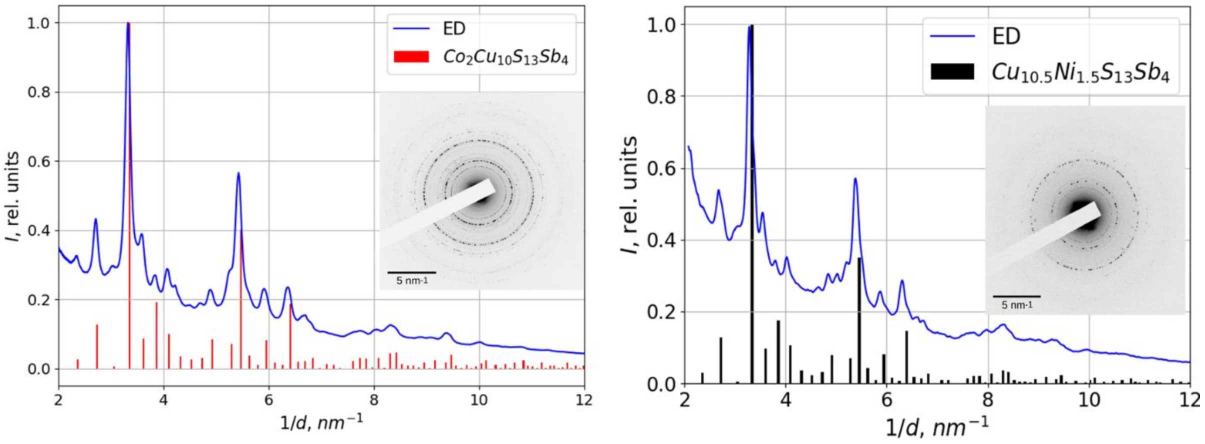

Figure S6: SAED pattern showing the presence of cubic phase for $Cu_{10}Co_2Sb_4S_{13}$ and $Cu_{10.5}Ni_{1.5}Sb_4S_{13}$ NCs.

5 min.

Element	Weight%	Atomic%
S K	27.91	48.62
Cu K	42.49	38.08
Zn K	2.50	0.61
Sb L	28.09	13.89
Totals	100.00	

10 min.

Element	Weight%	Atomic%
S K	27.91	48.62
Cu K	40.49	36.08
Zn K	3.50	2.41
Sb L	28.09	12.89
Totals	100.00	

15 min.

Element	Weight%	Atomic%
S K	26.38	46.86
Cu K	35.29	31.63
Zn K	8.86	7.72
Sb L	29.47	13.79
Totals	100.00	

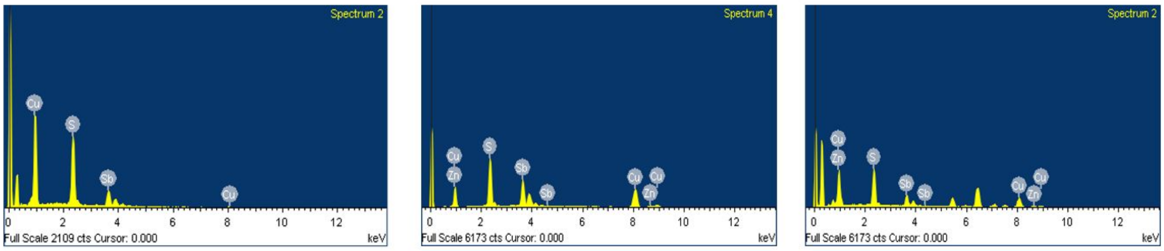

Figure S7: EDX elemental data $Cu_{10}Zn_2Sb_4S_{13}$ (CAZS) aliquot for 5, 10 and 15 min. at 240 ˚C.

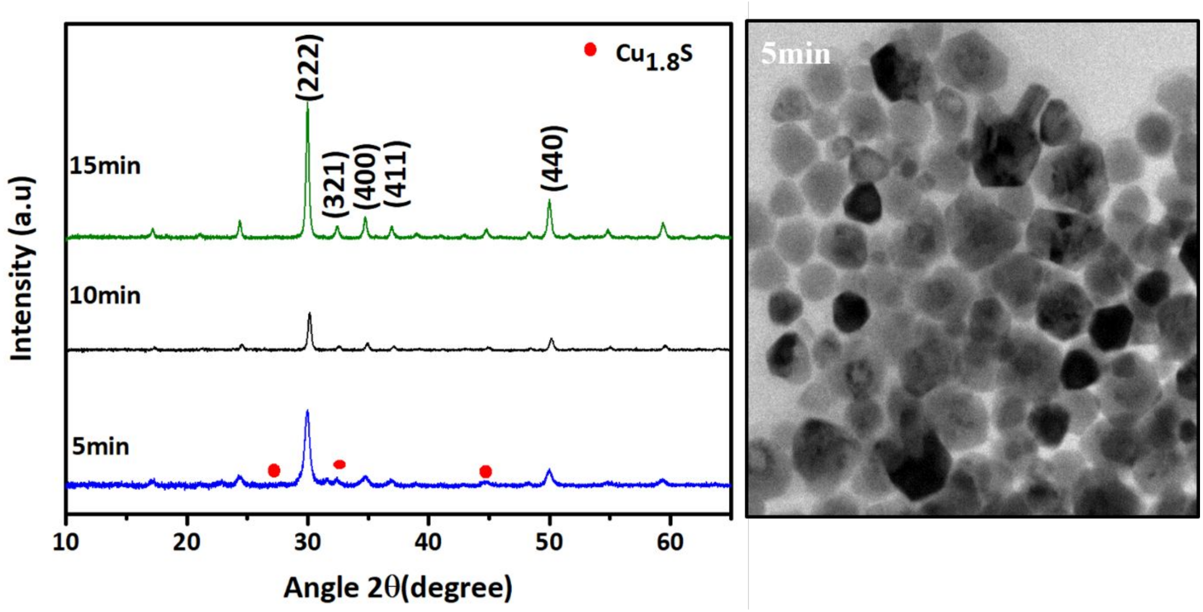

Figure S8: (a) XRD spectra of aliquots collected at different reaction time (5, 10, and 15min) for $Cu_{10.5}Ni_{1.5}Sb_4S_{13}$ with TEM images at different reaction time (scale bar for TEM images is 100nm).

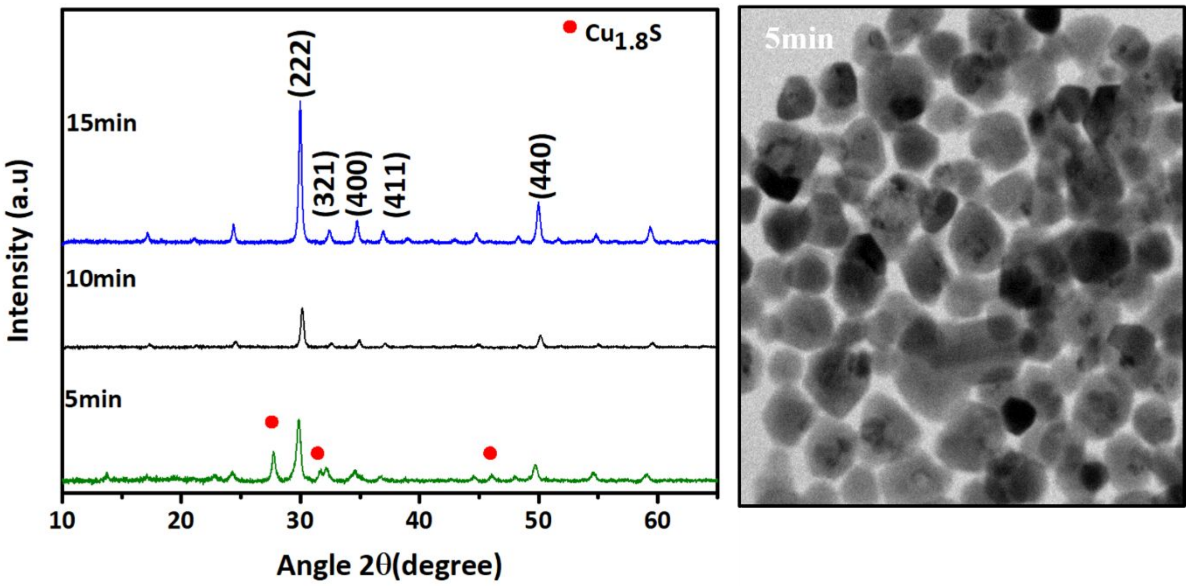

Figure S9: (a) XRD spectra of aliquots collected at different reaction time (5, 10, and 15min) for $Cu_{10}Co_2Sb_4S_{13}$ with TEM images at different reaction time (scale bar for TEM images is 100nm).

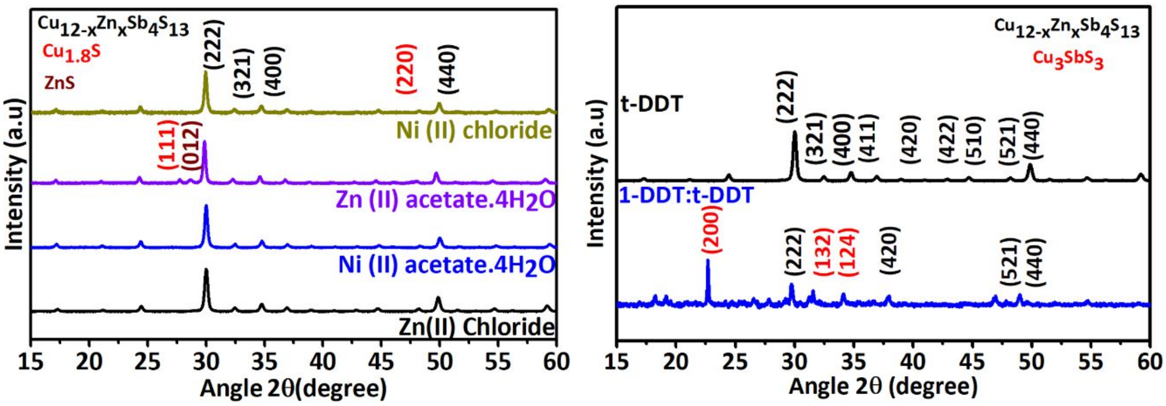

Figure S10: (a) XRD patterns of Cu_{10} $Zn_2Sb_4S_{13}$ and $Cu_{10.5}Sb_4Ni_{1.5}S_{13}$ NCs synthesize by reacting Cu $(OAc)_2$, with $NiCl_2$ or Zn $(OAC)_2.4H_2O$ keeping other condition constant ($SbCl_3$ with t-DDT in mixture of OLA and ODE. (b) XRD spectra of $Cu_{10}Zn_2Sb_4S_{13}$ NCs with different Sulphur sources (1-DDT and t-DDT).

For optimization of the reaction protocol, two different thiol-based S sources, 1-DDT and 3-DDT were used. Contrary to the previous reports on the formation of pure tetrahedrite phases using 1-DDT as sulphur source, the use of 1-DDT as a S source in the present study results in the formation of $Cu_{10-x}Sb_4M_xS_{13}$ as a minor phase and famatanite (Cu_3SbS_3) as a major phase **(figure S10b)**.[1, 2] Replacing 1-DDT with t-DDT allowed for a more rapid supply of sulphide ions which triggered fast and mature growth of **$Cu_{10}Zn_2Sb_4S_{13}$** nanostructures.[3]

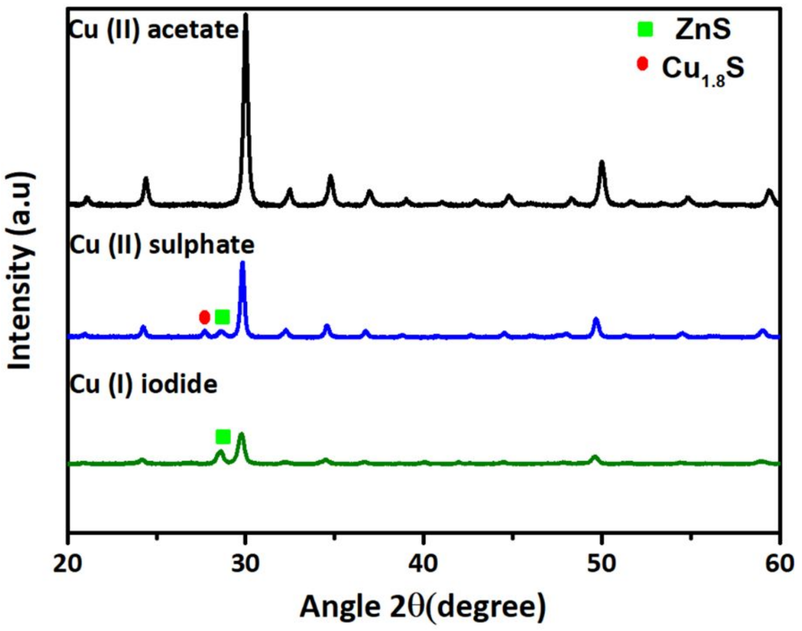

Figure S11. XRD patterns of controlled experiments using copper precursors of different reactivity Cu (I) iodide, Cu (II)sulphate and Cu (II) acetate with $ZnCl_2$ and $SbCl_3$ using t-DDT as a S source.

We explored three different Cu sources (Cu(I)iodide, Cu (II) Sulphate and Cu (II) acetate). The Cu_{10-x} Sb_4 M_xS_{13} was the major phase in all three cases t with the formation of $Cu_{1.8}S$ and metallic Sb as a side product for Cu (I) Iodide and Cu (II) sulphate, respectively **(figure S11)**. According to HSAB theory Cu^{II} is a harder acid than Cu^{I} and I is a soft Lewis acid than OAc^{2-} and SO_3^{4-}. All together, these considerations point at following reactivity trends for copper precursor: CuI > Cu $(OAc)_2$ > Cu(II)Sulphate.[4] Copper precursor of intermediate reactivity results in the formation of pure cubic Cu_{10-x} Sb_4 M_xS_{13} phase without any side product.

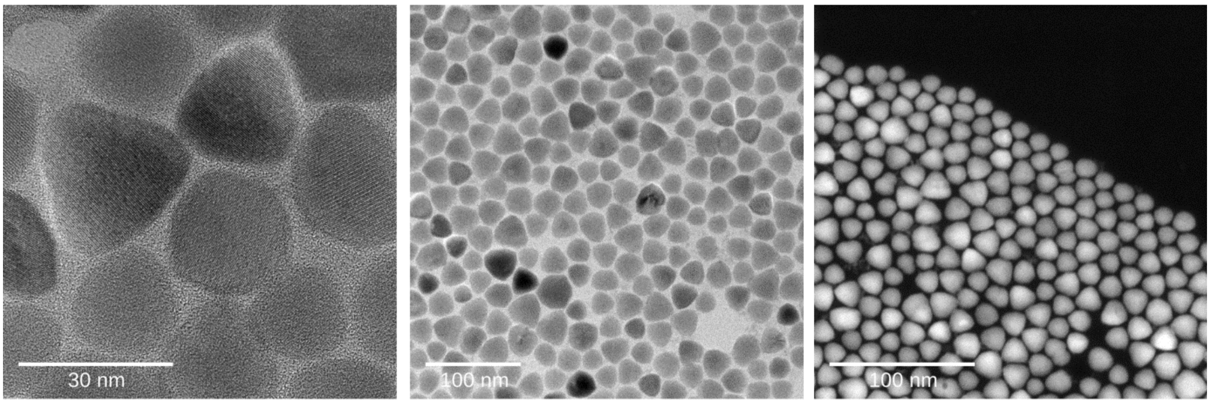

Figure S12.TEM images of $Cu_{10}Zn_2Sb_4S_{13}$ NCs at different magnification.

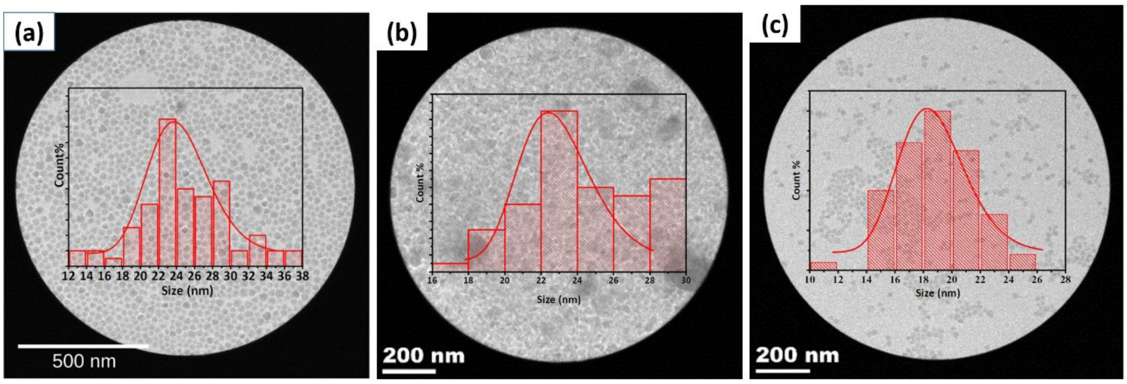

Figure S13: TEM images of (a) $Cu_{10}Zn_2Sb_4S_{13}$ (b) $Cu_{10.5}Ni_{1.5}Sb_4S_{13}$ and $Cu_{10}Co_2Sb_4S_{13}$ NCs.

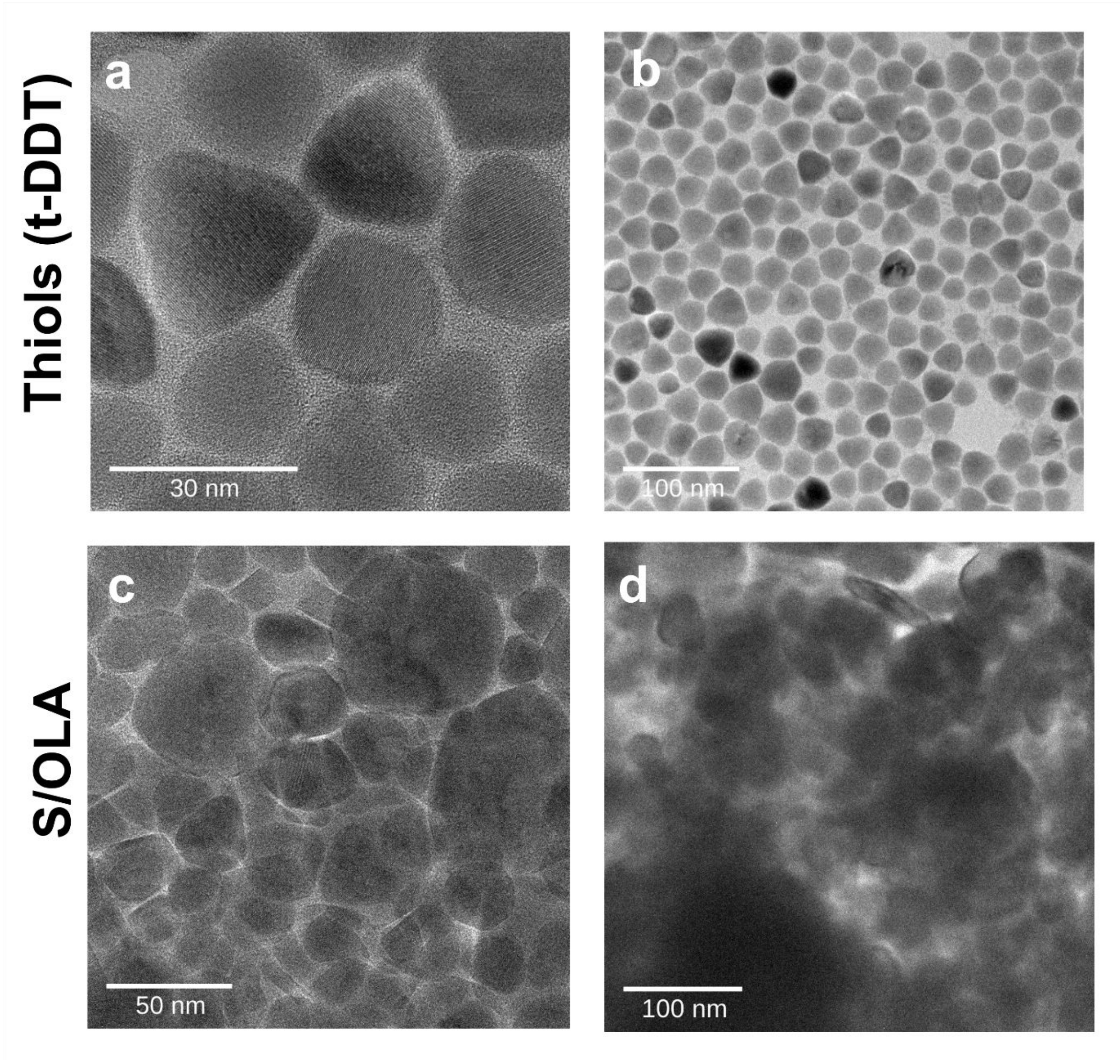

Figure S14: TEM images of $Cu_{10}Zn_2Sb_4S_{13}$ NCs synthesize using (a,b) thiols and (c,d) sulfur powder (S/OLA) as a sulfur precursor.

Synthesis of $Cu_{12}Sb_4S_{13}$ Nanocrystals:

In a typical synthesis 0.3 mmol of $Cu(CH_3COO)_2$, and 0.8 mmol of $Sb(Cl)_3$ with 4:1 ODE/OLA ratio by volume were added in a 25 mL three neck flask under Ar atmosphere and connected to a Schlenk line via a condenser. The reaction mixture was evacuated at 120 °C for 40 minutes to form a clear metal-ligand complex and to remove any moisture content. After this, the solution was purged with Ar and the temperature raised to 240 °C and 5 mmol of t-DDT swiftly

injected, which turned the solution color into black color. The reaction was then allowed to proceed for 15 min at 240 °C with continuous stirring for the growth of the nanocrystals. The reaction was terminated by removing the heating mantle and the dark colloidal solution was allowed to cool down to 100 °C before quenching with 5 mL of anhydrous toluene. The obtained nanocrystals were isolated from the solution by centrifugation and to effectively purify the nanocrystals the mother liquor was divided into two 50 mL centrifuge tubes dispersed in 10 mL of hexane, and centrifuged at 5000 rpm for 3 min. After that the supernatant was discarded while the residue was redispersed in hexane and acetone (3:1 v/v) and centrifuged at 3000 rpm for 5 min. The semi pure nanocrystals were then purified by several precipitation and dispersion cycles using chloroform/acetone/hexane (1:1:2 v/v). The washed NCs were vacuum dried in an oven at 70 ˚C overnight before measurement of the thermoelectric properties. The cleaned and dried nanoparticles were annealed at 490 °C and then pressed in to pellets by using a hot press technique. All the measurements were done at temperatures below 490 °C.

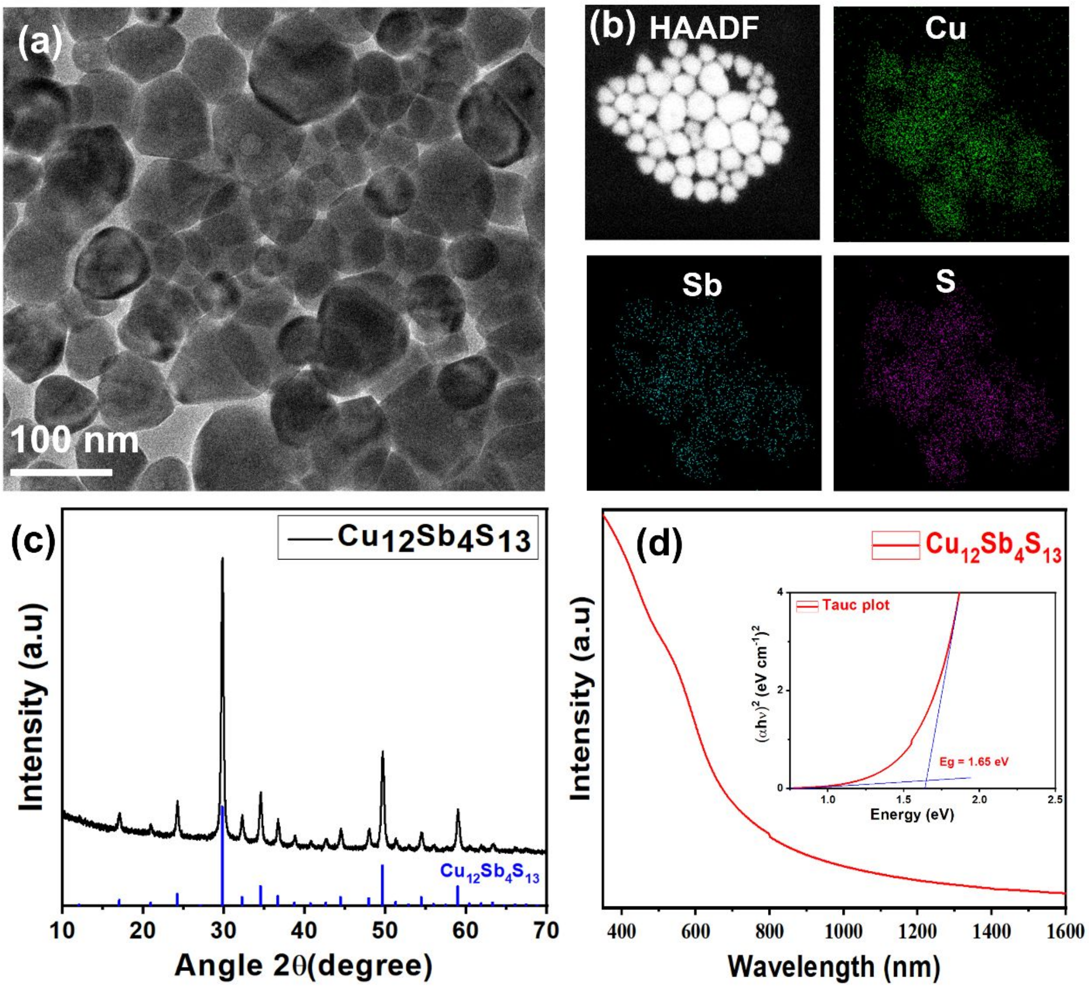

Figure S15. (a) TEM image of cubic phase $Cu_{12}Sb_4S_{13}$ NCs. (b) HAADF-STEM image of a $Cu_{12}Sb_4S_{13}$ NCs with corresponding STEM-EDS elemental maps related to Cu (green), Sb (blue), and S (purple) of $Cu_{12}Sb_4S_{13}$ NCs with (c) XRD pattern $Cu_{12}Sb_4S_{13}$ NCs and (d) UV–VIS absorbance spectra of $Cu_{12}Sb_4S_{13}$ NCs.

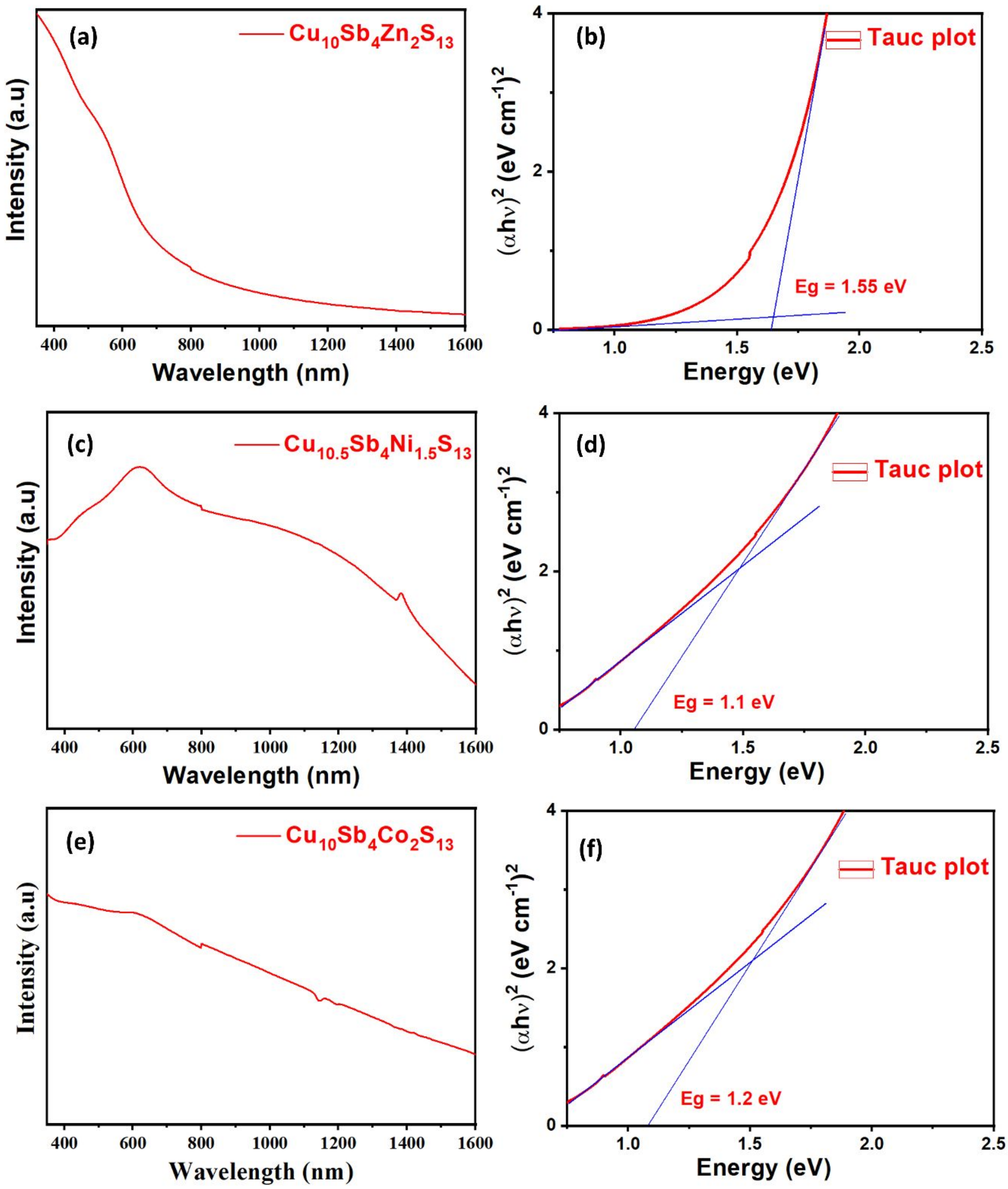

Figure S16: (a, c and e) Optical absorption spectra of $Cu_{10}Zn_2Sb_4S_{13}$, $Cu_{10.5}Ni_{1.5}Sb_4S_{13}$, and $Cu_{10}Co_2Sb_4S_{13}$ NCs and (b, d and f) corresponding Tauc plot to calculate bandgap of NCs.

To investigate the obtained $Cu_{10}Zn_2Sb_4S_{13}$, $Cu_{10.5}Ni_{1.5}Sb_4S_{13}$, and $Cu_{10}Co_2Sb_4S_{13}$ NCs optical characteristics, We first analysed UV–visible absorption at room temperature **(figures S15(a, c and e)**. The optical bandgap of the as-prepared NCs was determined from the acquired

absorption spectra. Extrapolating the straight-line section of the curve $(\alpha hv)^2$ versus energy (eV) **(figure S15(b, d and f)** for zero absorption coefficient value yields the bandgap energy E_g of $Cu_{10}Zn_2Sb_4S_{13}$, $Cu_{10.5}Ni_{1.5}Sb_4S_{13}$, and $Cu_{10}Co_2Sb_4S_{13}$ NCs were determined to be 1.5eV, 1.1eV and 1.2 eV which is lower than the reported value of pure tetrahedrite (1.7 eV).[1]

Table S2: Measured electrical and optical properties of substituted tetrahedrite.

Compositions	Resistivity (Ωcm)	Band gap (eV)
$Cu_{10}Zn_2Sb_4S_{13}$	10	1.5
$Cu_{10.5}Ni_{1.5}Sb_4S_{13}$	0.001	1.1
$Cu_{10}Co_2Sb_4S_{13}$	0.01	1.2

Additional References:

1. Liang, Q., et al., *Synthesis of Cu–Sb–S nanocrystals: insight into the mechanism of composition and crystal phase selection.* CrystEngComm, 2016. **18**(20): p. 3703-3710.
2. Ramasamy, K., et al., *Selective nanocrystal synthesis and calculated electronic structure of all four phases of copper–antimony–sulfide.* Chemistry of Materials, 2014. **26**(9): p. 2891-2899.
3. Bera, S., et al., *Predominated thermodynamically controlled reactions for suppressing cross nucleations in formation of multinary substituted tetrahedrite nanocrystals.* The Journal of Physical Chemistry Letters, 2018. **9**(8): p. 1907-1912.
4. Mantella, V., et al., *Colloidal synthesis of Cu–M–S (M= V, Cr, Mn) nanocrystals by tuning the copper precursor reactivity.* Chemistry of Materials, 2020. **32**(22): p. 9780-9786.
